# Prevalence and determinants of unmet healthcare needs in Chinese residents: evidence from the China family panel studies, 2012–2022

**DOI:** 10.3389/fpubh.2026.1925306

**Published:** 2026-09-16

**Authors:** Xinyi Jin, Xueying Lan, Shuang Ma

**Affiliations:** School of Management, Beijing University of Chinese Medicine, Beijing, China

**Keywords:** Chinese residents, healthcare-seeking behavior, prevalence, risk factors, unmet healthcare needs

## Abstract

**Introduction:**

To examine temporal trends in unmet healthcare needs among Chinese residents from 2012 to 2022, identify their influencing factors, and high-risk populations, and provide empirical evidence for optimizing health service allocation and relevant policy formulation.

**Methods:**

Based on six waves of the China Family Panel Studies (CFPS) spanning 2012 to 2022, this study included 11,067 residents aged 16 years and older who reported illness within the preceding 2 weeks, forming an unbalanced panel dataset with 30,925 observations. Guided by Andersen’s Behavioral Model of Health Services Use, generalized estimating equations (GEE) and panel fixed-effects logit regression models were employed to identify factors associated with unmet healthcare needs.

**Results:**

The prevalence of unmet healthcare needs among Chinese residents fluctuated significantly over the decade. It declined steadily from 37.50% in 2012 to 28.94% in 2016 and subsequently rebounded to 46.07% in 2022. Regression results indicated that employed and urban residents, were more vulnerable to unmet healthcare needs. Stronger health beliefs and higher trust in physicians served as crucial protective factors, whereas perceived disease severity and chronic disease management significantly shaped residents’ healthcare-seeking behavior.

**Conclusion:**

Unmet healthcare needs remain prevalent among Chinese residents and are affected by multidimensional risk factors. Future health policies should strengthen health system resilience. They must also deliver targeted interventions for high-risk groups, such as employed and urban residents. Furthermore, it is essential to promote public health education and foster physician–patient trust. Finally, scaling up standardized chronic disease management will facilitate early health screening and encourage proactive health management.

## 1 Introduction

Universal Health Coverage (UHC) has become a central issue in global health and a shared development goal to which governments worldwide are committed. It refers to a situation in which all members of society can equitably access high-quality health services they need without incurring financial risk when using them (1). China has continuously deepened its health system reform, which is closely aligned with the principles of UHC. In particular, following the comprehensive implementation of the “Healthy China 2030” strategy, a series of reform achievements have received widespread recognition from the international community. However, the period 2019–2022 coincided with the COVID-19 pandemic, which placed extreme strain on healthcare resources and substantially limited the capacity of health systems to deliver essential services. This disrupted access to routine non-COVID healthcare, potentially contributing to the rebound in unmet healthcare needs with long-term consequences. Unmet healthcare needs are defined as the gap between the healthcare services deemed necessary to address a specific health problem and those actually received by an individual (2, 3). This gap directly reflects the barriers individuals face in accessing healthcare and serves as a key negative indicator of healthcare accessibility and utilization equity (4). Its prolonged accumulation may lead to multiple adverse consequences, including deterioration in individual health (5), household financial risk (6), and exacerbation of societal health inequities (7). Compared with positive indicators of actual service utilization, such as outpatient and hospitalization rates, investigating unmet healthcare needs is more effective at directly identifying weaknesses in the supply and demand for health services from the problem end, thereby facilitating the targeted optimization of health policies.

The concept of unmet healthcare needs has been shaped by two paradigms. The clinical assessment paradigm relies on professional judgment to objectively determine whether an individual received appropriate care, but is limited to conditions with clear clinical guidelines. The subjective self-report paradigm, more common in population studies (8), captures individuals’ reports of needing but failing to obtain healthcare services (7). Building on these foundational paradigms, scholars have further classified unmet healthcare needs into five categories: (1) unperceived unmet needs; (2) subjective, voluntary unmet needs; (3) subjective, involuntary unmet needs; (4) subjective, clinically validated unmet needs; and (5) subjectively unmet expectations of care (7) (Figure 1). The present study focuses primarily on subjective unmet healthcare needs, including healthcare services that were denied, delayed, or forgone.

**Figure 1:**
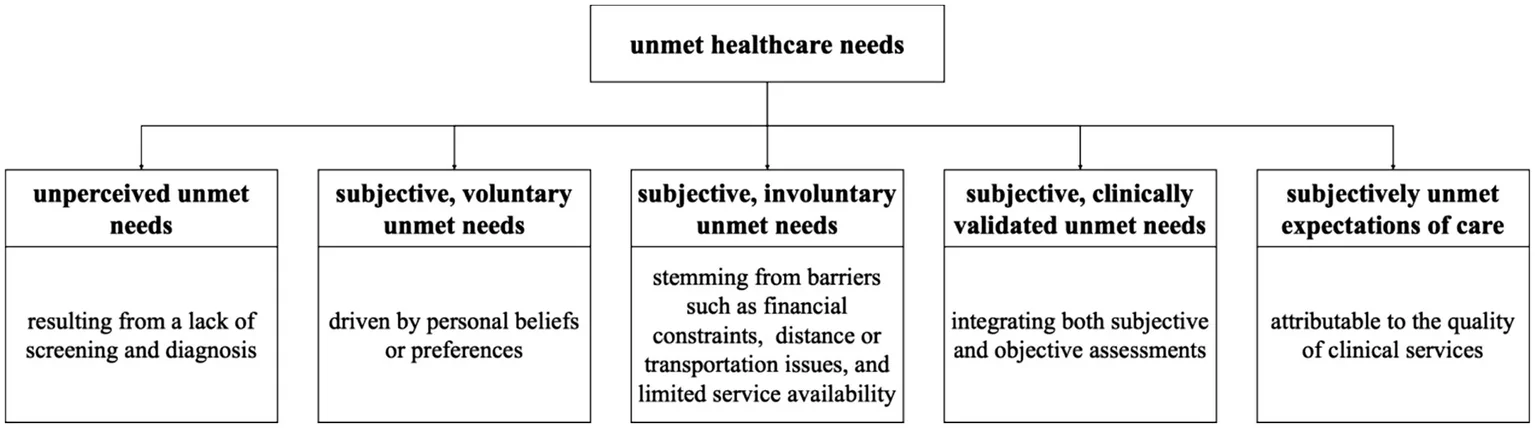
Common classification of unmet healthcare needs.

Existing studies on subjective unmet healthcare needs have largely been conducted in Europe, North America, the Asia-Pacific region, and the Middle East. Study populations have commonly focused on specific age groups or vulnerable subgroups. Existing evidence suggests substantial inequalities in unmet healthcare needs across population subgroups and regions (41, 42). 41% of unemployed individuals in Finland experienced unmet healthcare needs during 2022–2023 (9); among European migrant populations, the rate was 27.8% in 2013–2015 (10); and a systematic review estimated that approximately 10.4% of older adults globally had unmet healthcare needs (11). During the COVID-19 pandemic, a survey across 21 countries found that about 28.2 and 42.1% of respondents reported unmet preventive care needs and chronic disease care needs, respectively (12). Unmet healthcare needs remain pronounced among Chinese residents. In Jiangsu Province, 12.86, 2.22, and 48.89% of middle-aged and older adults reported unmet needs for outpatient services, inpatient care, and health check-ups in 2008, 2013, and 2018, respectively (13). In Shanghai in 2018, the non-utilization rate of outpatient services among migrants was 44.43%, higher than the 37.95% among non-migrants (14). Nationwide, 11.3% of middle-aged and older adults were unable to access needed medical services during the COVID-19 pandemic in 2020 (15). Studies have identified that common causes of unmet healthcare needs stem from both the supply and demand sides. At the healthcare system level, the main obstacles are limited service availability, long wait times, and inconvenient appointment scheduling. At the individual or household level, relevant barriers include accessibility constraints (e.g., lack of time, long travel distances, inconvenient transportation, mobility limitations, insufficient information), affordability barriers (financial hardship), and individual perceptions and preferences (e.g., viewing the illness as mild, lacking trust in the healthcare system) (16, 17). Furthermore, demographic, socioeconomic, and health-related characteristics, including age, sex, income, occupation, and self-rated health status, have been linked to unmet healthcare needs (9, 15, 18). Nevertheless, most prior studies rely on cross-sectional data, yielding inconsistent findings and failing to capture dynamic changes over time. Longitudinal evidence regarding the decade-long trends and time-varying determinants of unmet healthcare needs in China remains scarce, especially evidence explaining the post-pandemic rebound in unmet medical needs.

This study therefore utilized 10-year nationally representative panel data to characterize temporal trends in subjective unmet healthcare needs from 2012 to 2022. Guided by the Andersen health service utilization model, we further identified key predisposing, enabling, and need factors. The findings provide longitudinal evidence for optimizing health resource allocation and advancing equitable and sustainable UHC in China.

## 2 Materials and methods

### 2.1 Theoretical model

This study adopts the Behavioral Model of Health Services Use (the Andersen Model) as its theoretical framework. The model is widely recognized as an authoritative framework in health services research for examining the determinants of health service utilization and access to care. It was originally developed in 1968 by Ronald Max Andersen, an American medical sociologist, to explain variations in families’ use of health care services. The model has since evolved, with the individual as the primary unit of analysis, and has undergone five major revisions by 2013, reflecting its ongoing refinement and theoretical expansion (19–21).

As the core component of the Andersen Model, individual characteristics encompass both objective and subjective factors that influence healthcare utilization and comprise three dimensions: predisposing characteristics, enabling resources, and need factors. Predisposing characteristics are attributes that influence individuals’ healthcare-seeking decisions before illness onset and are not directly related to health service utilization, such as demographic characteristics, social structure, and health beliefs. Enabling resources denote individuals’ capacity to obtain healthcare services and the availability of resources, both of which indirectly affect utilization. This dimension encompasses financial and organizational resources. Need refers to an individual’s requirement for medical care, regarded as the prerequisite, core driver, and direct cause of health service utilization. It consists of perceived need (a subjective judgment of personal health status) and evaluated need (an objective measurement and professional assessment by physicians).

### 2.2 Data resource and samples

The data used in this study were drawn from the China Family Panel Studies (CFPS), conducted by the Institute of Social Science Survey (ISSS) at Peking University. The CFPS is a large-scale, nationally representative longitudinal social survey that captures socioeconomic, demographic, health-related, and educational changes in China through long-term, systematic data collection. Spanning 25 provinces, autonomous regions, and municipalities, the CFPS comprises community, household, adult, and child questionnaires. Its comprehensive content and high-quality data provide a robust empirical foundation for academic research and public policy analysis. Since its baseline survey was formally launched in 2010, the CFPS has conducted full-sample follow-up surveys every other year. To date, eight waves of national surveys have been completed, and publicly available data have been released through 2022 (22).

This study used six waves of longitudinal panel data from the CFPS spanning 2012 to 2022. Because children’s healthcare-seeking behavior is largely determined by parents, lacks the autonomy of adults, and has distinct determinants, this group has been examined in prior specialized studies. Accordingly, consistent with the CFPS sample design, this study focused on respondents aged 16 years and older. The analysis sample was limited to residents who self-reported illness or physical discomfort within 2 weeks before each survey wave. After data cleaning and variable harmonization, individuals with at least two observations were retained, resulting in an unbalanced panel dataset. The final analytical sample comprised 11,067 individuals and 30,925 person-wave observations. The numbers of observations in 2012, 2014, 2016, 2018, 2020, and 2022 were 4,462, 6,024, 6,295, 6,395, 4,103, and 3,646, respectively (Figure 2).

**Figure 2:**
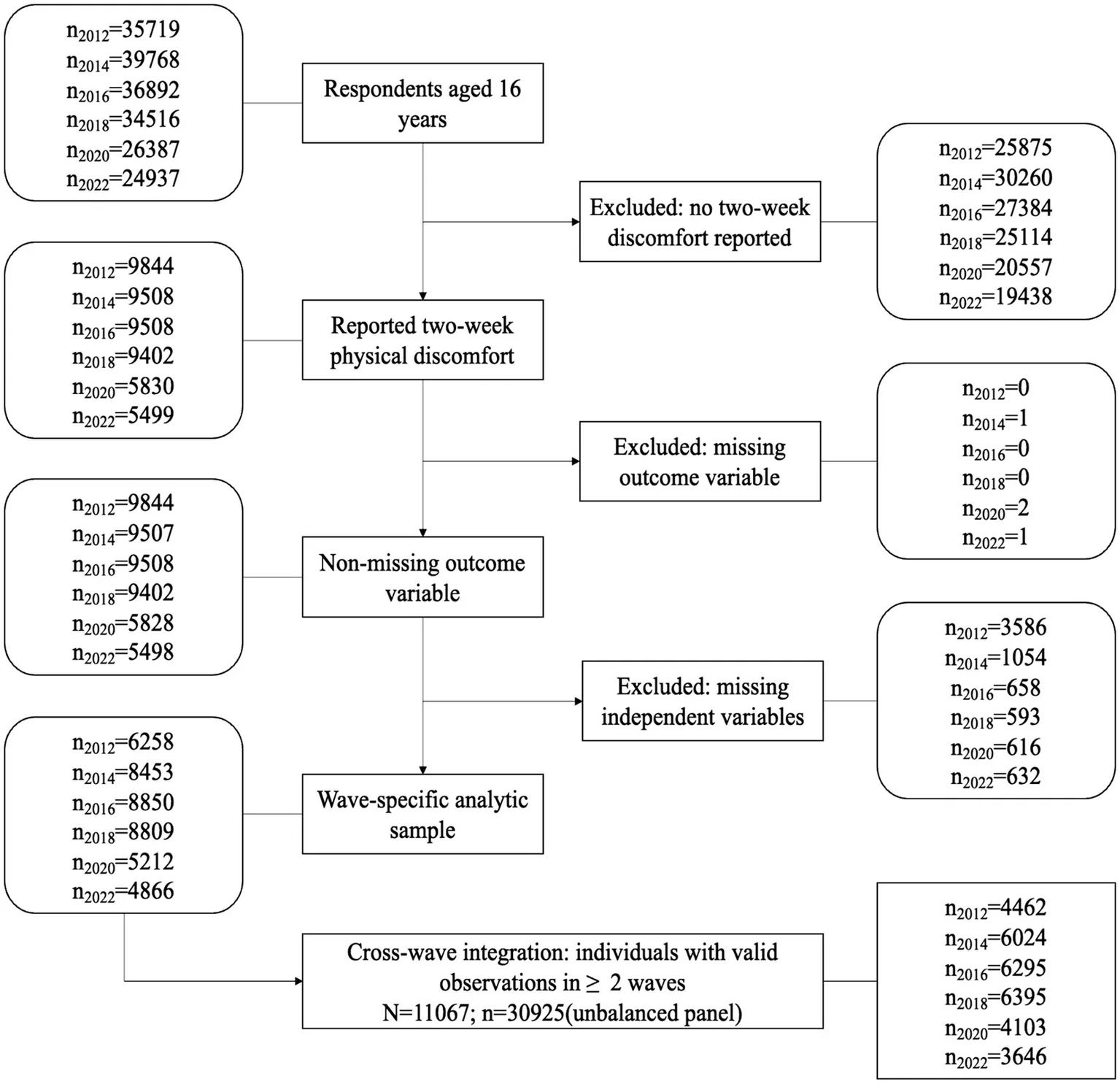
Wave-by-wave sample selection flowchart, CFPS 2012–2022.

### 2.3 Measures

#### 2.3.1 Dependent variable

The outcome variable in this study is subjectively perceived unmet healthcare needs. Based on the CFPS questionnaire items and established research practices, this variable is defined as whether residents sought medical consultation for physical discomfort within 2 weeks prior to the interview. Specifically, it is derived from responses to two questions: “Have you experienced physical discomfort in the past 2 weeks?” and “Did you see a doctor for your illness in the past 2 weeks?” Respondents who reported illness in the preceding 2 weeks and sought medical care were classified as having no unmet healthcare needs and assigned a value of 0. Those who were ill but did not seek care were classified as having unmet healthcare needs and assigned a value of 1.

#### 2.3.2 Independent variables

Drawing on relevant domestic and international literature and the CFPS questionnaire, this study initially identified potential determinants of unmet healthcare needs among urban and rural residents in China (Supplementary Tables S8 to S13). Guided by the Andersen Model, these factors were grouped into three domains: predisposing, enabling, and need factors. The variables and their coding schemes are presented in Table 1.

**Table 1:** Variable coding.

Variable type	Variable name	Coding scheme
Dependent variable	Healthcare needs	0 = met, 1 = unmet
Independent variables		
Predisposing factors	Gender	0 = female, 1 = male
	Age	1 = 16–25, 2 = 26–35, 3 = 36–45, 4 = 46–55, 5 = 56–65, 6 = 66 or above
	Marital status	1 = never married, 2 = married (having a spouse), 3 = cohabitation, 4 = divorced, 5 = widowed
	Education	1 = primary school or below, 2 = junior high school, 3 = senior high school/secondary school/technical school/vocational senior school, 4 = 3-year college, 5 = 4-year college or above
	Household registration status (hukou)	0 = Agricultural, 1 = Non-agricultural (including resident hukou)
	Employment status	0 = Non-employed (unemployed/not in labor force), 1 = Employed
	Health beliefs	1 = very low, 2 = low, 3 = fair, 4 = high
	Trust in doctors	1 = low, 2 = fair, 3 = high
	Medical service satisfaction	1 = very unsatisfied, 2 = unsatisfied, 3 = fair, 4 = satisfied, 5 = very satisfied
Enabling factors	Per capita household income percentile	1 = low, 2 = low-middle, 3 = middle-high, 4 = high
	Health insurance type	1 = Uninsured, 2 = Public medical insurance, 3 = Urban employee Basic Medical Insurance, 4 = Basic medical insurance for urban and rural residents
	Region	1 = Eastern China, 2 = Central China, 3 = Western China, 4 = Northeastern China
	Residence	0 = rural, 1 = urban
	Familysize (members)	1 = 1, 2 = 2, 3 = 3–4, 4 = 5 or more
Need factors	Self-rated health	1 = excellent, 2 = very good, 3 = good, 4 = fair, 5 = poor
	Perceived symptom severity	1 = not serious, 2 = fair, 3 = serious
	Number of diagnosed chronic disease	1 = 0, 2 = 1, 3 = 2 or more

### 2.4 Statistical analysis

Descriptive analyses were conducted to characterize the study population and examine unmet healthcare needs across groups with varying characteristics. Categorical variables were summarized with frequencies and percentages. In addition, line and bar charts were used to illustrate overall patterns and temporal trends in unmet healthcare needs among Chinese residents.

For prevalence estimation, the CFPS wave-specific individual cross-sectional weight (rswt_natcs) was applied to ensure national representativeness, and age- and sex-standardized prevalence was calculated by direct standardization using the 2020 Seventh National Population Census as the common reference population (Supplementary Tables S1, S4). Regression results are reported unweighted, with weighted GEE, weighted survey logit, and IPW-GEE models serving as sensitivity analyses, which yielded consistent results (Supplementary Tables S2, S3, S7).

Because the dataset is multi-wave longitudinal panel data derived from the CFPS, measurements of the same individual across years tend to be correlated, violating the independence assumption of conventional statistical tests, including the chi-square test (23). Therefore, we adopted generalized estimating equations (GEE) for multivariable analyses. This approach was used to examine the statistical significance of temporal trends in overall unmet healthcare needs among Chinese residents between 2012 and 2022, as well as to provide the overall average associations of influencing factors. We fitted generalized estimating equation (GEE) models with a binomial distribution, a logit link, and an exchangeable working correlation structure, clustering on individual identifiers; robust sandwich standard errors were used. An autoregressive AR (1) working correlation structure was examined as a sensitivity analysis (Supplementary Tables S17).

Using CFPS data from 2012 to 2022, this study constructed an unbalanced panel dataset to examine the determinants among Chinese residents, Because the dependent variable is binary, coded 0 for satisfied needs and 1 for unmet needs, a panel logit model was employed. The estimation strategy was selected from among pooled logit regression, random-effects, and fixed-effects models. First, the applicability of pooled regression was verified by testing for the presence of individual effects ( $\mu_{i}$ ). The statistically significant result (*p* < 0.05) indicated that the pooled logit specification was inappropriate, suggesting that either a random-effects (RE) or fixed-effects (FE) model was applicable. Second, the choice between RE and FE models depended on whether individual effects ( $\mu_{i}$ ) were correlated with the explanatory variables. If no such correlation exists, the RE model would be preferred; otherwise, the FE model would be more appropriate. The Hausman test yielded a significant result (*p* < 0.05), rejecting the null hypothesis of consistent RE estimates and favoring the FE model. Furthermore, fixed-effects estimators are widely favored in empirical studies because they can effectively control for all unobserved, time-invariant confounding factors (24). Therefore, following the above model selection procedure, this study used panel fixed-effects logit regression to examine factors of within-person variation in unmet healthcare needs, while controlling for time-invariant unobserved confounders via individual fixed effects from 2012 to 2022.

## 3 Results

### 3.1 Characteristics of study samples

The final analytical sample comprised residents aged 16 or older who reported physical discomfort in at least two survey waves between 2012 and 2022, yielding 30,925 person-wave observations. The distribution across waves was 4,462 (2012), 6,024 (2014), 6,295 (2016), 6,395 (2018), 4,103 (2020), and 3,646 (2022). Females accounted for 57.08 to 60.79% of the sample across survey waves. The share of young adults aged 16–25 decreased from 4.21 to 1.18%, while the share of older adults aged 66 and older increased from 8.36 to 24.71%. Married individuals (with a spouse) accounted for 83.80 to 89.74%. The share of individuals with a primary school education or less declined from 62.30 to 48.68%, while the share with a bachelor’s degree or higher increased from 2.53 to 5.87%. The proportion of non-agricultural hukou holders rose from 19.61 to 27.48%. The “fair” level of health beliefs was consistently the most prevalent category, whereas the “very low” level remained the least common. The proportion of respondents reporting a high level of trust in physicians ranged from 50.74 to 58.49%. The proportion of residents who were satisfied or very satisfied with the conditions at the healthcare facility they usually visited increased from 53.38 to 72.63%. The lowest-income group accounted for 25.10–32.50% of the sample. Health insurance coverage rose from 91.57 to 93.80%. Residents from western China accounted for 32.98 to 35.15%, while the share of rural residents declined from 64.72 to 51.65%. Those who self-rated their health as “unhealthy” ranged from 40.74 to 44.04%. The combined proportion perceiving their illness severity as “fair” or “serious” ranged from 77.43 to 85.48%. The proportion of respondents with physician-diagnosed chronic diseases ranged from21.34 to 39.63% (Table 2).

**Table 2:** Basic characteristics of the study participants (%).

**Variables**	**2012** **(*N* = 4,462)**	**2014** **(*N* = 6,024)**	**2016** **(*N* = 6,295)**	**2018** **(*N* = 6,395)**	**2020** **(*N* = 4,103)**	**2022** **(*N* = 3,646)**
Predisposing factors						
Gender: female	57.08	60.79	60.11	60.30	58.66	58.72
Age						
16–25	4.21	3.50	3.05	2.22	2.05	1.18
26–35	10.40	9.86	10.72	11.27	12.23	10.89
36–45	24.63	17.02	13.82	12.92	12.26	13.08
46–55	28.48	25.98	25.97	25.82	27.44	23.67
56–65	23.91	26.73	25.73	23.94	23.37	26.47
66+	8.36	16.92	20.70	23.83	22.64	24.71
Marital status						
Never married	4.35	3.95	3.99	4.07	4.61	4.00
Married (having a spouse)	89.74	86.09	85.12	83.80	84.18	83.87
Cohabitation	0.31	0.43	0.49	0.56	0.51	0.27
Divorced	1.32	1.78	2.00	2.11	2.92	3.40
Widowed	4.28	7.75	8.40	9.46	7.77	8.45
Education						
Primary school or below	62.30	62.32	60.92	58.20	51.13	48.68
Junior high school	21.94	22.18	22.49	23.82	26.83	27.26
Senior high school/secondary school/technical school/vocational senior school	9.66	9.91	10.36	10.40	12.23	12.89
3-year college	3.56	3.40	3.62	4.25	5.00	5.29
4-year college or above	2.53	2.19	2.61	3.33	4.80	5.87
Hukou: non-agricultural	19.61	25.18	24.53	23.67	25.32	27.48
Employment status: employed	98.34	71.91	69.07	70.62	72.02	69.42
Health beliefs	17.60	17.36	16.84	15.61	19.34	17.97
Very low	6.30	5.48	5.13	4.50	5.07	4.47
Low	20.26	18.01	17.47	16.00	19.67	18.16
Fair	52.44	56.16	53.57	51.34	58.18	59.49
High	21.00	20.35	23.83	28.16	17.09	17.88
Trust in doctors	38.75	32.59	34.93	35.45	32.52	34.98
Low	11.54	10.51	11.15	11.42	8.87	10.64
Fair	37.72	32.27	35.19	35.40	32.63	35.93
High	50.74	57.22	53.66	53.18	58.49	53.43
Healthcare service satisfaction	8.22	7.29	8.30	10.71	8.81	10.52
Very unsatisfied	0.81	0.86	0.95	2.13	2.02	2.39
Unsatisfied	8.70	7.25	8.48	10.88	9.09	10.64
Fair	37.11	40.85	35.74	22.33	17.50	14.34
Satisfied	49.48	45.40	48.98	55.70	61.03	59.93
Very satisfied	3.90	5.63	5.85	8.96	10.36	12.70
Enabling factors	25.80	26.17	31.16	31.11	30.46	31.83
*Per Capita* household Income percentile	26.00	26.00	27.53	28.49	28.11	27.93
Low	25.10	26.49	31.68	31.26	30.93	32.50
Low-middle	26.98	26.33	27.55	28.66	28.00	28.50
Middle-high	27.25	23.56	23.43	22.36	23.32	22.79
High	20.66	23.62	17.35	17.72	17.74	16.21
Health insurance type	30.43	28.42	28.46	29.57	28.89	28.23
Uninsured	8.43	6.64	7.28	6.90	7.60	6.20
Public medical insurance	2.49	2.59	1.70	2.22	2.17	1.48
UEBMI (urban employee)	9.41	11.94	12.09	12.60	12.55	16.65
URRBMI (urban–rural resident)	79.67	78.83	78.94	78.28	77.67	75.67
Region						
Eastern China	27.95	27.89	28.24	28.80	28.59	27.40
Central China	24.79	23.71	23.76	22.14	23.35	23.20
Western China	34.16	32.98	33.88	35.15	34.24	33.85
Northeastern China	13.11	15.42	14.11	13.90	13.82	15.55
Residence: urban	35.28	43.56	45.04	45.43	46.89	48.35
Family size	46.47	40.73	41.33	42.41	41.01	40.20
1 person	1.46	3.70	4.50	4.94	5.09	6.12
2 persons	15.53	19.37	19.46	21.74	21.52	23.86
3–4 persons	42.94	38.00	35.92	34.31	33.83	32.42
5+ persons	40.07	38.93	40.13	39.01	39.56	37.60
Need factors	75.84	61.43	61.92	63.08	64.10	61.22
Self-rated health						
Excellent	1.70	3.45	2.92	3.47	3.27	2.85
Very good	5.15	6.51	5.05	4.55	5.14	5.18
Good	25.57	29.52	26.83	31.15	34.22	37.16
Fair	23.53	19.79	23.08	17.51	15.57	13.30
Poor	44.04	40.74	42.11	43.32	41.80	41.50
Perceived symptom severity						
Not serious	22.57	17.68	17.81	14.53	18.25	17.58
Fair	39.85	46.48	40.86	42.13	51.04	52.44
Serious	37.58	35.84	41.33	43.35	30.71	29.98
Number of chronic diseases						
0	78.66	61.94	61.40	62.55	63.66	60.37
1	15.58	22.51	20.05	17.70	16.72	16.92
2 or more	5.76	15.55	18.55	19.75	19.62	22.71

### 3.2 Prevalence of unmet healthcare needs

Overall, the prevalence of unmet healthcare needs among Chinese residents showed marked fluctuations over the study period. The weighted unmet need rate declined from 36.20% in 2012 to a nadir of 21.72% in 2016, followed by a sustained rebound to 38.45% in 2022. After age standardization, the prevalence followed a similar trajectory but with notably larger amplitude: decreasing from 37.50% in 2012 to 28.94% in 2016, then rising sharply to 46.07% in 2022. Both the weighted and standardized rates reached their lowest points in 2016 and their highest points in 2022. The standardized rate consistently exceeded the weighted rate across all survey waves, and the disparity widened over time—from 1.30 percentage points in 2012 to 7.62 percentage points in 2022—suggesting that demographic shifts partly obscured the true magnitude of unmet healthcare needs. GEE analysis indicated that, compared with 2012, the odds of unmet healthcare needs were significantly lower in 2014 (OR = 0.752, *p* < 0.001), 2016 (OR = 0.638, *p* < 0.001), and 2018 (OR = 0.603, *p* < 0.001), whereas the odds were significantly higher in 2020 (OR = 1.152, *p* = 0.005) and 2022 (OR = 1.379, *p* < 0.001) (Table 3 and Figure 3).

**Table 3:** GEE analysis of unmet healthcare needs among Chinese residents, 2012–2022.

Variable	Category	Unweighted OR (95%CI)	*P*
Gender	Female (ref)	1 (ref)	
	Male	0.952 (0.882–1.027)	0.204
Age	16–25 (ref)	1 (ref)	
	26–35	0.860 (0.720–1.027)	0.096
	36–45	0.790 (0.656–0.951)	**0.013**
	46–55	0.705 (0.585–0.849)	**<0.001**
	56–65	0.574 (0.475–0.694)	**<0.001**
	66+	0.523 (0.427–0.640)	**<0.001**
Marital status	Never married (ref)	1 (ref)	
	Married	0.789 (0.672–0.926)	**0.004**
	Cohabitation	1.045 (0.677–1.614)	0.841
	Divorced	0.814 (0.641–1.034)	0.091
	Widowed	0.891 (0.729–1.089)	0.261
Education	Primary or below (ref)	1 (ref)	
	Junior high	1.017 (0.940–1.101)	0.67
	Senior high/technical	1.177 (1.057–1.311)	**0.003**
	3-year college	1.341 (1.136–1.583)	**<0.001**
	4-year college or above	1.529 (1.275–1.833)	**<0.001**
Hukou	Agricultural (ref)	1 (ref)	
	Non-agricultural	1.110 (1.014–1.215)	**0.024**
Employment	Non-employed (ref)	1 (ref)	
	Employed	1.172 (1.083–1.268)	**<0.001**
Health beliefs	Very low (ref)	1 (ref)	
	Low	0.855 (0.748–0.977)	**0.022**
	Fair	0.699 (0.611–0.800)	**<0.001**
	High	0.575 (0.497–0.666)	**<0.001**
Trust in doctors	Low (ref)	1 (ref)	
	Fair	0.796 (0.726–0.873)	**<0.001**
	High	0.745 (0.679–0.816)	**<0.001**
Healthcare satisfaction	Very unsatisfied (ref)	1 (ref)	
	Unsatisfied	1.010 (0.804–1.269)	0.929
	Fair	0.969 (0.778–1.207)	0.78
	Satisfied	0.823 (0.662–1.022)	0.078
	Very satisfied	0.734 (0.578–0.933)	**0.012**
Income	Low (ref)	1 (ref)	
	Low-middle	0.967 (0.897–1.042)	0.381
	Middle-high	0.982 (0.905–1.065)	0.656
	High	1.022 (0.930–1.123)	0.657
Health insurance	Uninsured (ref)	1 (ref)	
	Public medical care	0.838 (0.673–1.044)	0.115
	UEBMI	0.897 (0.784–1.026)	0.112
	URRBMI	0.831 (0.747–0.924)	**<0.001**
Region	Eastern (ref)	1 (ref)	
	Central	0.884 (0.812–0.963)	**0.005**
	Western	0.916 (0.844–0.994)	**0.034**
	Northeastern	1.493 (1.356–1.643)	**<0.001**
Residence	Rural (ref)	1 (ref)	
	Urban	1.220 (1.138–1.307)	**<0.001**
Family size	1 (ref)	1 (ref)	
	2	1.037 (0.881–1.220)	0.661
	3–4	0.937 (0.800–1.099)	0.425
	≥5	0.893 (0.760–1.049)	0.168
Self-rated health	Excellent (ref)	1 (ref)	
	Very good	1.066 (0.890–1.277)	0.487
	Good	1.063 (0.910–1.242)	0.441
	Fair	0.987 (0.840–1.159)	0.869
	Poor	0.788 (0.672–0.924)	**0.003**
Symptom severity	Not serious (ref)	1 (ref)	
	Fair	0.630 (0.587–0.675)	**<0.001**
	Serious	0.339 (0.312–0.368)	**<0.001**
Chronic diseases	0 (ref)	1 (ref)	
	1	0.452 (0.417–0.490)	**<0.001**
	≥2	0.401 (0.364–0.441)	**<0.001**
Year	2012 (ref)	1 (ref)	
	2014	0.752 (0.688–0.823)	**<0.001**
	2016	0.638 (0.582–0.700)	**<0.001**
	2018	0.603 (0.549–0.663)	**<0.001**
	2020	1.152 (1.043–1.272)	**0.005**
	2022	1.379 (1.241–1.531)	**<0.001**

Bolded values indicate that *p* < 0.05.

**Figure 3:**
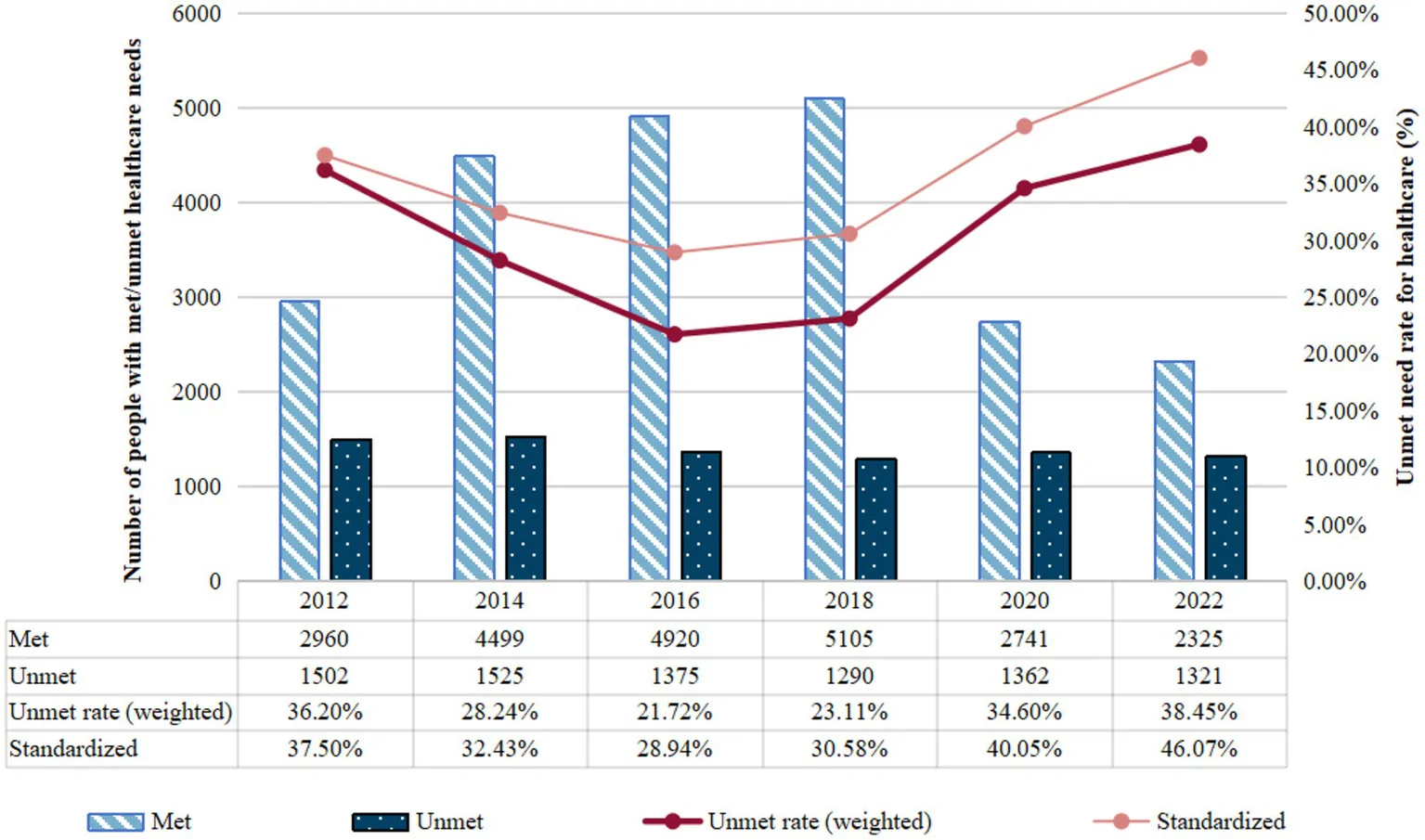
Prevalence of unmet healthcare needs among Chinese residents, 2012–2022.

### 3.3 Factors associated with unmet healthcare needs

All variables in this study are binary or multi-class variables. Collinearity diagnostics indicate that the GVIF^(1/(2*Df)) values are all less than 2, indicating that there are no serious multicollinearity issues among the variables (Supplementary Tables S15, S16).

Trends in unmet healthcare needs across subgroups largely mirrored the overall pattern, with an initial decline followed by an increase, and 2016 as the turning point. Regarding predisposing factors, the GEE results indicated that the risk of unmet healthcare needs decreased progressively with age, being lowest among individuals aged 56–65 years (OR = 0.574, *p* < 0.001) and 66 years and older (OR = 0.523, *p* < 0.001) relative to those aged 16–25 years. Married individuals had a lower risk than those who had never married (OR = 0.789, *p* = 0.004), whereas other marital statuses did not differ significantly. The risk increased with educational attainment, reaching OR = 1.529 (*p* < 0.001) among those with 4-year college or above, and was also higher among non-agricultural hukou holders (OR = 1.110, *p* = 0.024) and employed individuals (OR = 1.172, *p* < 0.001).

Stronger health beliefs (high: OR = 0.575, *p* < 0.001), greater trust in doctors (high: OR = 0.745, *p* < 0.001), and greater satisfaction with medical care (very satisfied: OR = 0.734, *p* = 0.012) were associated with a lower risk. Among enabling factors, urban residents (OR = 1.220, *p* < 0.001) and residents of northeastern China (OR = 1.493, *p* < 0.001) faced a higher risk, whereas residents of central (OR = 0.884, *p* = 0.005) and western (OR = 0.916, *p* = 0.034) China had a lower risk; income and family size were not statistically significant. Among health insurance types, only URRBMI coverage was significantly protective (OR = 0.831, *p* < 0.001).

Regarding need factors, poorer self-rated health (OR = 0.788, *p* = 0.003), higher perceived symptom severity (serious: OR = 0.339, *p* < 0.001), and diagnosed chronic diseases (one: OR = 0.452; two or more: OR = 0.401, both *p* < 0.001) were associated with a markedly lower risk (Table 3).

Panel fixed-effects logit regression, which exploits within-person variation over time and controls for all time-invariant individual characteristics (Supplementary Tables S14), indicated that older age, being employed, urban residence, and UEBMI coverage were associated with a higher likelihood of forgoing medical care following illness onset, whereas higher educational attainment, stronger health beliefs, greater trust in doctors, poorer self-rated health, higher perceived symptom severity, and diagnosed chronic diseases were associated with a lower likelihood.

Compared with young adults aged 16–25 years, residents aged 36 years and older faced a significantly higher risk of unmet healthcare needs within individuals, with the risk progressively decreasing with age (36–45 years: OR = 1.63, *p* = 0.03; 46–55 years: OR = 2.08, *p* = 0.01; 56–65 years: OR = 2.05, *p* = 0.02; 66 years and older: OR = 2.27, *p* = 0.02). In contrast, higher educational attainment was associated with significantly lower odds among those with 3-year college (OR = 0.39, *p* = 0.01) or 4-year college or above (OR = 0.29, *p* < 0.001). Employed individuals (OR = 1.26, *p* < 0.001), urban residents (OR = 1.34, *p* = 0.01), and UEBMI enrollees (OR = 1.26, *p* = 0.05) faced significantly higher risks, whereas marital status, hukou, region, income, family size, and healthcare satisfaction showed no significant within-person associations. Compared with individuals reporting very low health beliefs, those with fair (OR = 0.64, *p* < 0.001) or high (OR = 0.57, *p* < 0.001) health beliefs had a significantly lower risk, and fair (OR = 0.80, *p* < 0.001) and high (OR = 0.76, *p* < 0.001) levels of trust in doctors were similarly protective.

Regarding need factors, compared with individuals who self-rated their health as “excellent,” those reporting “poor” health had a significantly lower risk (OR = 0.72, *p* = 0.02). Compared with the “not serious” reference group, respondents who perceived their illness as “fair” had a 42.0% lower risk (OR = 0.58, *p* < 0.001), and those who perceived it as “serious” had a 72.0% lower risk (OR = 0.28, *p* < 0.001). Having one diagnosed chronic disease (OR = 0.50, *p* < 0.001) or two or more chronic diseases (OR = 0.47, *p* < 0.001) was likewise associated with approximately halved odds relative to having no chronic conditions. Regarding survey year, the odds of unmet healthcare needs were significantly lower in 2014 (OR = 0.66, *p* < 0.001), 2016 (OR = 0.48, *p* < 0.001), and 2018 (OR = 0.45, *p* < 0.001) than in 2012, whereas the odds in 2020 (OR = 0.95, *p* = 0.54) and 2022 (OR = 1.07, *p* = 0.51) did not differ significantly (Table 4).

**Table 4:** Panel fixed-effects logit regression analysis of unmet healthcare needs among Chinese residents, 2012–2022.

Variable	Category	Unweighted OR (95%CI)	*P*
Age	16–25 (ref)	1 (ref)	
	26–35	1.21 (0.85–1.70)	0.29
	36–45	1.63 (1.04–2.56)	**0.03**
	46–55	2.08 (1.25–3.49)	**0.01**
	56–65	2.05 (1.14–3.74)	**0.02**
	66+	2.27 (1.15–4.48)	**0.02**
Marital status	Never married (ref)	1 (ref)	
	Married	1.08 (0.70–1.67)	0.71
	Cohabitation	1.46 (0.71–3.03)	0.30
	Divorced	0.96 (0.54–1.68)	0.88
	Widowed	1.00 (0.57–1.75)	0.99
Education	Primary or below (ref)	1 (ref)	
	Junior high	0.81 (0.58–1.13)	0.21
	Senior high/technical	0.72 (0.41–1.28)	0.27
	3-year college	0.39 (0.19–0.79)	**0.01**
	4-year college or above	0.29 (0.12–0.66)	**<0.001**
Hukou	Agricultural (ref)	1 (ref)	
	Non-agricultural	0.95 (0.72–1.25)	0.69
Employment	Non-employed (ref)	1 (ref)	
	Employed	1.26 (1.11–1.43)	**<0.001**
Health beliefs	Very low (ref)	1 (ref)	
	Low	0.85 (0.67–1.06)	0.15
	Fair	0.64 (0.50–0.83)	**<0.001**
	High	0.57 (0.43–0.74)	**<0.001**
Trust in doctors	Low (ref)	1 (ref)	
	Fair	0.80 (0.69–0.92)	**<0.001**
	High	0.76 (0.64–0.88)	**<0.001**
Healthcare satisfaction	Very unsatisfied (ref)	1 (ref)	
	Unsatisfied	1.13 (0.83–1.56)	0.43
	Fair	1.07 (0.79–1.45)	0.67
	Satisfied	1.02 (0.75–1.38)	0.92
	Very satisfied	0.94 (0.67–1.31)	0.70
Income	Low (ref)	1 (ref)	
	Low-middle	1.02 (0.90–1.15)	0.75
	Middle-high	1.04 (0.90–1.19)	0.58
	High	1.01 (0.85–1.19)	0.92
Health insurance	Uninsured (ref)	1 (ref)	
	Public medical care	1.19 (0.83–1.73)	0.34
	UEBMI	1.26 (1.00–1.58)	**0.05**
	URRBMI	0.99 (0.84–1.16)	0.89
Region	Eastern (ref)	1 (ref)	
	Central	0.72 (0.43–1.21)	0.21
	Western	0.56 (0.29–1.08)	**0.09**
	Northeastern	0.74 (0.27–1.99)	0.55
Residence	Rural (ref)	1 (ref)	
	Urban	1.34 (1.07–1.67)	**0.01**
Family size	1 (ref)	1 (ref)	
	2	0.99 (0.73–1.35)	0.96
	3–4	0.92 (0.68–1.26)	0.62
	≥5	0.96 (0.69–1.33)	0.81
Self-rated health	Excellent (ref)	1 (ref)	
	Very good	1.09 (0.81–1.46)	0.57
	Good	1.01 (0.78–1.31)	0.93
	Fair	0.97 (0.74–1.26)	0.81
	Poor	0.72 (0.55–0.95)	**0.02**
Symptom severity	Not serious (ref)	1 (ref)	
	Fair	0.58 (0.52–0.65)	**<0.001**
	Serious	0.28 (0.25–0.32)	**<0.001**
Chronic diseases	0 (ref)	1 (ref)	
	1	0.50 (0.45–0.57)	**<0.001**
	≥2	0.47 (0.41–0.54)	**<0.001**
Year	2012 (ref)	1 (ref)	
	2014	0.66 (0.58–0.74)	**<0.001**
	2016	0.48 (0.42–0.55)	**<0.001**
	2018	0.45 (0.39–0.52)	**<0.001**
	2020	0.95 (0.81–1.13)	0.54
	2022	1.07 (0.88–1.28)	0.51

Bolded values indicate that *p* < 0.05.

Notably, the directions of the age and education associations were reversed relative to the GEE estimates, reflecting the distinction between within-person and population-averaged effects. Gender, being time-invariant, was absorbed by the fixed-effects estimator and is therefore not estimable in Table 4.

## 4 Discussion

### 4.1 Overall, unmet healthcare needs among Chinese residents showed a fluctuating pattern and persisted at a relatively high level

Our results revealed that the prevalence of unmet healthcare needs among Chinese residents needs stayed elevated across the entire study timeframe: it first declined and then rebounded.

Specifically, the prevalence dropped consistently from 37.50% in 2012 to 28.94% in 2016, and then rebounded slightly to 30.58% in 2018. While our figures were marginally higher than the data released in the Fifth and Sixth National Health Service Surveys, the overall downward trend was in line with previous evidence (25, 26). This finding reflects the phased outcomes of healthcare system reform implemented in China over this period. Nationwide universal health insurance was fully established during these years, with its coverage expanding continuously in both scope and depth, which gradually alleviated residents’ financial burdens associated with medical services (27). Meanwhile, the rollout of key policies, such as hierarchical medical systems and contracted family doctor services, combined with the ongoing expansion of primary healthcare facilities, has significantly enhanced the service capacity of grassroots medical institutions (28). Collectively, these efforts mitigated financial and geographical barriers to healthcare access and advanced health equity.

Nevertheless, the prevalence of unmet healthcare needs rose sharply to 40.05% in 2020 and further reached 46.07% in 2022. This rebound temporally coincided with the COVID-19 pandemic. During the pandemic, patients shifted toward online consultations and often delayed care due to fears of hospital-acquired infections (29, 30). Notably, while the GEE model indicated significantly elevated odds of unmet needs in 2020 (OR = 1.152, *P* = 0.005) and 2022 (OR = 1.379, *P* < 0.001), neither year remained significant in the fixed-effects model (both *p* > 0.5), indicating that the rebound operated mainly at the population level, through period-wide shocks such as pandemic-related service disruption together with shifts in sample composition, rather than as a persistent within-person deterioration in access. It is imperative to strengthen the resilience of health systems and their capacity to cope with various risks, so as to guarantee the sustained and stable provision of healthcare services.

For international reference, prior research reported that the proportion of adults with unmet healthcare needs ranged from 7 to 33% across 11 countries in 2016 (31), while the figure stood at 3.8% among the EU population aged 16 and above in 2024 (32). By comparison, the level of unmet healthcare needs among Chinese residents remains relatively high.

### 4.2 Employed and urban residents are the primary risk factors

Significant Non-employed-Employed and urban–rural disparities were observed in the risk of unmet healthcare needs. GEE analysis showed that employed residents were significantly more likely than their unemployed counterparts to report unmet healthcare needs (OR = 1.172, *p* < 0.001), and urban residents were significantly more likely than their rural counterparts to do so (OR = 1.220, *p* < 0.001). The conditional fixed-effects logit model yielded consistent results. This finding is consistent with the majority of previous studies (33, 34). One potential explanation is that urban residents, who generally exhibit higher health awareness, may have sought care prior to the two-week recall period and were already adhering to prescribed treatments. This, in turn, contributes to a higher proportion of respondents reporting no medical consultation during the reference period. Another potential explanation lies in the fast-paced, high-pressure urban lifestyle, which often imposes demanding work schedules and elevated stress on residents. When combined with healthcare-seeking barriers such as appointment difficulties, long wait times, and complex clinical procedures, these constraints may compel employed and urban residents to forgo treatment or resort to self-medication for mild, non-urgent health issues in order to minimize both time and economic costs. By contrast, the healthcare delivery system and care-seeking pathways in rural areas may be comparatively simpler, offering a more accessible care model for rural residents. It is vital to optimize healthcare service workflows for urban and employed populations and to mitigate non-financial barriers to accessing care. Specifically, the hierarchical medical system should be further promoted, and the service capacity of primary care facilities such as community health centers should be strengthened to achieve efficient patient triage. In parallel, evening and after-hours outpatient services should be expanded, so that employed individuals no longer postpone seeking care because of the high opportunity cost of taking time off work.

### 4.3 High health beliefs and strong trust in physicians stand as crucial protective factors against unmet healthcare needs

The GEE analysis showed that, compared to residents with very low health beliefs, those holding fair health beliefs experienced an approximately 14.5% lower risk of unmet needs (OR = 0.855, *p* = 0.022). Individuals with higher health beliefs saw a further, substantial reduction of 30.1% (OR = 0.699, *p* < 0.001). The conditional fixed-effects logit model yielded consistent results. Typically, people with stronger health beliefs possess greater health literacy, monitor their well-being more closely, take more proactive steps to seek care, and demonstrate better treatment adherence (35). Continuous systematic health education should be delivered to improve public health literacy and early disease recognition. Similarly, when compared to those reporting low trust in doctors, residents with fair trust had about a 20.4% lower risk of unmet needs (OR = 0.796, *p* < 0.001), while those with high trust benefited from an additional 25.5% risk reduction (OR = 0.745, *p* < 0.001). Physician-patient trust serves as the cornerstone of the therapeutic relationship, playing a pivotal role in shaping care-seeking behavior; its absence frequently results in poorer treatment adherence (36). Enhancing humanistic care and doctor-patient communication across healthcare settings also helps protect people’s mental wellbeing.

### 4.4 Perceived symptom severity and chronic disease management profoundly shape healthcare-seeking behavior

The GEE analysis results show that, first, the perceived severity of symptoms stands out as a pivotal driver of healthcare utilization. Residents who considered their symptoms “fairly serious” demonstrated an approximately 37.0% lower risk of unmet healthcare needs compared to those who viewed their symptoms as “not serious” (OR = 0.630, *p* < 0.001). Meanwhile, those who perceived their condition as “serious” saw an even more substantial reduction in risk—approximately 66.1% (OR = 0.339, *p* < 0.001). The logit model with panel fixed effects also shows the same results. These findings align consistently with prior research (37, 38). Subjective appraisal of the threat posed by illness is the most direct and potent motivator for seeking healthcare. When residents perceive a health problem as tolerable or negligible, they are more likely to forgo or delay seeking care (39).

Second, a higher number of diagnosed chronic disease was linked to more prompt healthcare-seeking behavior—a finding that aligns with prior evidence (40). Residents with one chronic condition demonstrated a 54.8% lower risk of unmet healthcare needs compared to those without (OR = 0.452, *p* < 0.001), and those with two or more chronic conditions showed an even greater reduction of 59.9% (OR = 0.401, *p* < 0.001). This likely stems from the greater proactive engagement of individuals managing chronic illnesses, who tend to seek care earlier to prevent disease progression and complications. At the same time, chronic disease management programs and health education provided by healthcare institutions may substantially contribute to fulfilling these patients’ healthcare demands. We can apply effective chronic disease management practices, including stable doctor-patient ties, regular follow-ups and targeted health education, to better meet residents’ health needs.

Several limitations should be acknowledged. First, regarding selective attrition, comparisons of retained and attrited respondents across waves (Supplementary Tables S5 and S6), together with inverse-probability-weighted GEE models, suggested that our findings were largely robust to attrition related to observed characteristics; however, residual bias from dropout related to unobserved factors cannot be excluded. Second, our outcome captures subjectively perceived unmet healthcare needs: the analysis was conditional on self-reported illness, and individuals with objective care needs who did not perceive or report physical discomfort were not captured, so that our estimates reflect perceived rather than total unmet need. Third, the opposing directions of the age and education coefficients between the GEE and fixed-effects models warrant cautious interpretation: because age, period, and cohort are perfectly collinear in panel data, these components cannot be fully disentangled, and we therefore interpret both patterns as reflecting cohort succession at the between-person level and individual change at the within-person level, rather than as pure age or education effects. Finally, this study preliminarily analyzed regional disparities in residents’ unmet healthcare needs. However, due to data availability constraints, regional-level healthcare resource and policy indicators could not be incorporated, and their potential impacts warrant further investigation in future research.

## 5 Conclusion

This study systematically assessed the trends and influencing factors of unmet healthcare needs among Chinese residents from 2012 to 2022. The results showed a fluctuating pattern over the decade. The overall rate of unmet needs first decreased from 37.50% in 2012 to 28.94% in 2016, then increased to 46.07% in 2022, remaining at a relatively high level. Unmet healthcare needs were influenced by multiple factors. Employed and urban residents, were more likely to report unmet needs. High health literacy and trust in physicians served as key protective factors. Perceived disease severity and chronic disease management drove care-seeking behavior. Future efforts should focus on strengthening health system resilience, prioritizing high-risk populations, enhancing health education and patient–physician trust, and leveraging chronic disease management to promote proactive health engagement.

## Data Availability

The original contributions presented in the study are included in the article/Supplementary material, further inquiries can be directed to the corresponding author/s.
