# Supplementary material for: Prevalence and determinants of unmet healthcare needs in Chinese residents: evidence from the China family panel studies, 2012–2022
Source: Front Public Health. 2026 Sep 16;14:1925306. doi: 10.3389/fpubh.2026.1925306 (PMC13624089; doi:10.3389/fpubh.2026.1925306)
Supplement: Supplementary file 1 [file Supplementary_file_1.docx]

**Supplementary Appendix**

Prevalence and determinants of unmet healthcare needs in Chinese residents: Evidence from the China Family Panel Studies (CFPS), 2012-2022

Xinyi Jin, Xueying Lan, Shuang Ma*

**Contents**

[Supplementary Table S1: Weighted and Unweighted Crude Prevalence of Unmet Healthcare Needs among Chinese Residents, 2012–2022	1](#_Toc19477)

[Supplementary Table S2: Weighted GEE Analysis of Unmet Healthcare Needs Among Chinese Residents Aged 16 and Older, 2012–2022	2](#_Toc15245)

[Supplementary Table S3: Weighted Survey Logit Regression Analysis of Factors Associated with Unmet Healthcare Needs among Chinese Residents, 2012–2022	5](#_Toc10184)

[Supplementary Table S4: Age–Sex Standardized Prevalence of Unmet Healthcare Needs among Chinese Residents, 2012–2022	8](#_Toc29214)

[Supplementary Table S5: Wave-to-wave transitions of the analytic sample, CFPS 2012–2022	9](#_Toc26936)

[Supplementary Table S6: Preceding-wave characteristics of stayers and attriters, CFPS 2018-2020	11](#_Toc14346)

[Supplementary Table S7: Unweighted vs IPW-weighted estimates from multivariable GEE models	14](#_Toc19429)

[Supplementary Table S8: Classification of Household Registration Types	17](#_Toc31054)

[Supplementary Table S9: Classification of Employment status	18](#_Toc14421)

[Supplementary Table S10: Classification of Physical Exercise	19](#_Toc24305)

[Supplementary Table S11: Classification of Health Insurance	20](#_Toc21433)

[Supplementary Table S12: Classification of Region	20](#_Toc16507)

[Supplementary Table S13: Variable Coding	22](#_Toc18964)

[Supplementary Table S14: Intra-group Variation	24](#_Toc27160)

[Supplementary Table S15: Collinearity Diagnosis	25](#_Toc3665)

[Supplementary Table S16: Urban-Rural Year Interaction	26](#_Toc9420)

[Supplementary Table S17: Sensitivity analysis of GEE with AR(1) correlation structure	27](#_Toc26648)

**Supplementary Table S1: Weighted and Unweighted Crude Prevalence of Unmet Healthcare Needs among Chinese Residents, 2012–2022**

| Year | N  (unweighted) | N  (weighted) | Crude unweighted % | 95% CI  (unweighted) | Crude weighted % | 95% CI  (weighted) |
| --- | --- | --- | --- | --- | --- | --- |
| 2012 | 4462 | 4388 | 33.66 | (32.28 - 35.05) | 36.20 | (33.60 - 38.79) |
| 2014 | 6024 | 5813 | 25.32 | (24.22 - 26.41) | 28.24 | (25.96 - 30.52) |
| 2016 | 6295 | 6029 | 21.84 | (20.82 - 22.86) | 21.72 | (19.86 - 23.58) |
| 2018 | 6395 | 6078 | 20.17 | (19.19 - 21.16) | 23.11 | (21.12 - 25.09) |
| 2020 | 4103 | 3866 | 33.20 | (31.75 - 34.64) | 34.60 | (31.70 - 37.50) |
| 2022 | 3646 | 3413 | 36.23 | (34.67 - 37.79) | 38.45 | (35.65 - 41.25) |

**Supplementary Table S2: Weighted GEE Analysis of Unmet Healthcare Needs Among Chinese Residents Aged 16 and Older, 2012–2022**

| **Variable** | **β** | **OR** | **95%**  **CI(OR)** | **95%**  **CI(OR)** | **p** |
| --- | --- | --- | --- | --- | --- |
| **Predisposing factors** |  |  |  |  |  |
| **Gender (ref.: female)** |  |  |  |  |  |
| male | -0.06 | 0.94 | -0.18 | 0.05 | 0.26 |
| **Age (ref.: 16-25)** |  |  |  |  |  |
| 26-35 | 0.01 | 1.01 | -0.31 | 0.33 | 0.97 |
| 36-45 | -0.13 | 0.88 | -0.45 | 0.19 | 0.42 |
| 46-55 | -0.19 | 0.83 | -0.51 | 0.13 | 0.24 |
| 56-65 | -0.42 | 0.66 | -0.74 | -0.09 | 0.01 |
| 66+ | -0.56 | 0.57 | -0.91 | -0.22 | 0.00 |
| **Marital status (ref.: never married)** |  |  |  |  |  |
| married (having a spouse) | -0.38 | 0.68 | -0.64 | -0.12 | 0.00 |
| cohabitation | -0.43 | 0.65 | -1.10 | 0.25 | 0.22 |
| divorced | -0.37 | 0.69 | -0.75 | 0.02 | 0.06 |
| widowed | -0.21 | 0.81 | -0.53 | 0.11 | 0.20 |
| **Education (ref.: primary school or below)** |  |  |  |  |  |
| junior high school | -0.04 | 0.96 | -0.15 | 0.08 | 0.52 |
| senior high school/secondary school/technical school/vocational senior school | 0.03 | 1.03 | -0.13 | 0.18 | 0.75 |
| 3-year college | 0.13 | 1.13 | -0.12 | 0.37 | 0.31 |
| 4-year college or above | 0.16 | 1.17 | -0.14 | 0.45 | 0.29 |
| **Hukou (ref.: agricultural)** |  |  |  |  |  |
| non-agricultural | 0.16 | 1.17 | 0.02 | 0.30 | 0.03 |
| **Employment status (ref.: non-employed)** |  |  |  |  |  |
| employed | 0.10 | 1.11 | -0.02 | 0.22 | 0.09 |
| **Health beliefs (ref.: very low)** |  |  |  |  |  |
| low | -0.09 | 0.91 | -0.28 | 0.10 | 0.33 |
| fair | -0.34 | 0.71 | -0.53 | -0.15 | 0.00 |
| high | -0.57 | 0.57 | -0.78 | -0.36 | 0.00 |
| **Trust in doctors (ref.: low)** |  |  |  |  |  |
| fair | -0.23 | 0.79 | -0.37 | -0.10 | 0.00 |
| high | -0.25 | 0.78 | -0.39 | -0.12 | 0.00 |
| **Healthcare service satisfaction (ref.: very unsatisfied)** |  |  |  |  |  |
| unsatisfied | -0.12 | 0.89 | -0.44 | 0.21 | 0.48 |
| fair | -0.14 | 0.87 | -0.45 | 0.17 | 0.38 |
| satisfied | -0.29 | 0.75 | -0.60 | 0.01 | 0.06 |
| very satisfied | -0.32 | 0.73 | -0.66 | 0.03 | 0.07 |
| **Enabling factors** |  |  |  |  |  |
| **Per capita household income percentile (ref.: low)** |  |  |  |  |  |
| low-middle | 0.01 | 1.01 | -0.11 | 0.12 | 0.90 |
| middle-high | -0.01 | 0.99 | -0.13 | 0.11 | 0.85 |
| high | 0.01 | 1.01 | -0.13 | 0.15 | 0.90 |
| **Health insurance type (ref.: uninsured)** |  |  |  |  |  |
| public medical insurance | -0.23 | 0.79 | -0.60 | 0.13 | 0.21 |
| UEBMI (urban employee) | -0.01 | 0.99 | -0.22 | 0.20 | 0.93 |
| URRBMI (urban-rural resident) | -0.16 | 0.85 | -0.32 | 0.00 | 0.05 |
| **Region (ref.: Eastern China)** |  |  |  |  |  |
| Central China | 0.03 | 1.04 | -0.09 | 0.15 | 0.57 |
| Western China | -0.01 | 0.99 | -0.13 | 0.12 | 0.93 |
| Northeastern China | 0.41 | 1.51 | 0.27 | 0.55 | 0.00 |
| **Residence (ref.: rural)** |  |  |  |  |  |
| urban | 0.11 | 1.11 | 0.01 | 0.21 | 0.04 |
| **Family size (ref.: 1 person)** |  |  |  |  |  |
| 2 persons | -0.02 | 0.98 | -0.26 | 0.23 | 0.89 |
| 3-4 persons | -0.09 | 0.91 | -0.33 | 0.15 | 0.47 |
| 5+ persons | -0.15 | 0.86 | -0.40 | 0.09 | 0.22 |
| **Need factors** |  |  |  |  |  |
| **Self-rated health (ref.: excellent)** |  |  |  |  |  |
| very good | -0.03 | 0.97 | -0.33 | 0.26 | 0.82 |
| good | 0.02 | 1.02 | -0.24 | 0.28 | 0.88 |
| fair | -0.13 | 0.88 | -0.40 | 0.14 | 0.33 |
| poor | -0.32 | 0.72 | -0.59 | -0.05 | 0.02 |
| **Perceived symptom severity (ref.: not serious)** |  |  |  |  |  |
| fair | -0.53 | 0.59 | -0.63 | -0.43 | 0.00 |
| serious | -1.21 | 0.30 | -1.33 | -1.08 | 0.00 |
| **Number of chronic diseases (ref.: 0)** |  |  |  |  |  |
| 1 | -0.80 | 0.45 | -0.92 | -0.68 | 0.00 |
| 2+ | -1.00 | 0.37 | -1.15 | -0.86 | 0.00 |
| **Survey year** |  |  |  |  |  |
| **Year (ref.: 2012)** |  |  |  |  |  |
| 2014 | -0.25 | 0.78 | -0.38 | -0.12 | 0.00 |
| 2016 | -0.37 | 0.69 | -0.49 | -0.24 | 0.00 |
| 2018 | -0.45 | 0.64 | -0.58 | -0.31 | 0.00 |
| 2020 | 0.25 | 1.29 | 0.10 | 0.40 | 0.00 |
| 2022 | 0.37 | 1.45 | 0.21 | 0.52 | 0.00 |
| Constant |  |  |  |  |  |
| (_cons) | 1.62 | 5.05 | 1.02 | 2.22 | 0.00 |

**Supplementary Table S3: Weighted Survey Logit Regression Analysis of Factors Associated with Unmet Healthcare Needs among Chinese Residents, 2012–2022**

| **变量 Variable** | **β** | **OR** | **95%**  **CI(OR)** | **95%**  **CI(OR)** | | **p** |
| --- | --- | --- | --- | --- | --- | --- |
| **Predisposing factors** |  |  |  |  |  | |
| **Gender (ref.: female)** |  |  |  |  |  | |
| male | -0.04 | 0.96 | -0.17 | 0.09 | 0.56 | |
| **Age (ref.: 16-25)** |  |  |  |  |  | |
| 26-35 | -0.07 | 0.93 | -0.43 | 0.29 | 0.70 | |
| 36-45 | -0.12 | 0.88 | -0.45 | 0.21 | 0.46 | |
| 46-55 | -0.22 | 0.80 | -0.55 | 0.11 | 0.19 | |
| 56-65 | -0.37 | 0.69 | -0.72 | -0.02 | 0.04 | |
| 66+ | -0.59 | 0.56 | -0.96 | -0.22 | 0.00 | |
| **Marital status (ref.: never married)** |  |  |  |  |  | |
| **married (having a spouse)** | -0.32 | 0.73 | -0.64 | 0.01 | 0.06 | |
| cohabitation | -0.32 | 0.73 | -0.99 | 0.36 | 0.36 | |
| divorced | -0.43 | 0.65 | -0.88 | 0.03 | 0.06 | |
| widowed | -0.02 | 0.98 | -0.34 | 0.31 | 0.92 | |
| **Education (ref.: primary school or below)** |  |  |  |  |  | |
| junior high school | -0.04 | 0.96 | -0.17 | 0.09 | 0.53 | |
| senior high school/secondary school/technical school/vocational senior school | 0.07 | 1.07 | -0.11 | 0.26 | 0.45 | |
| 3-year college | 0.22 | 1.24 | -0.07 | 0.50 | 0.13 | |
| 4-year college or above | 0.14 | 1.15 | -0.23 | 0.50 | 0.45 | |
| **Hukou (ref.: agricultural)** |  |  |  |  |  | |
| non-agricultural | 0.14 | 1.15 | -0.06 | 0.33 | 0.16 | |
| **Employment status (ref.: non-employed)** |  |  |  |  |  | |
| employed | 0.07 | 1.07 | -0.09 | 0.23 | 0.38 | |
| **Health beliefs (ref.: very low)** |  |  |  |  |  | |
| low | -0.07 | 0.93 | -0.31 | 0.17 | 0.55 | |
| fair | -0.27 | 0.76 | -0.52 | -0.02 | 0.03 | |
| high | -0.55 | 0.58 | -0.79 | -0.31 | 0.00 | |
| **Trust in doctors (ref.: low)** |  |  |  |  |  | |
| fair | -0.25 | 0.78 | -0.42 | -0.09 | 0.00 | |
| high | -0.27 | 0.76 | -0.44 | -0.09 | 0.00 | |
| **Healthcare service satisfaction (ref.: very unsatisfied)** |  |  |  |  |  | |
| unsatisfied | -0.25 | 0.78 | -0.75 | 0.25 | 0.32 | |
| fair | -0.16 | 0.85 | -0.66 | 0.34 | 0.53 | |
| satisfied | -0.34 | 0.71 | -0.83 | 0.15 | 0.17 | |
| very satisfied | -0.34 | 0.71 | -0.83 | 0.15 | 0.18 | |
| **Enabling factors** |  |  |  |  |  | |
| **Per capita household income percentile (ref.: low)** |  |  |  |  |  | |
| low-middle | 0.02 | 1.02 | -0.13 | 0.17 | 0.76 | |
| middle-high | 0.03 | 1.03 | -0.14 | 0.21 | 0.71 | |
| high | 0.06 | 1.06 | -0.13 | 0.25 | 0.54 | |
| **Health insurance type (ref.: uninsured)** |  |  |  |  |  | |
| public medical insurance | -0.11 | 0.90 | -0.54 | 0.32 | 0.63 | |
| UEBMI (urban employee) | -0.03 | 0.97 | -0.28 | 0.23 | 0.84 | |
| URRBMI (urban-rural resident) | -0.18 | 0.83 | -0.39 | 0.03 | 0.09 | |
| **Region (ref.: Eastern China)** |  |  |  |  |  | |
| Central China | 0.04 | 1.04 | -0.18 | 0.25 | 0.74 | |
| Western China | -0.02 | 0.98 | -0.24 | 0.20 | 0.88 | |
| Northeastern China | 0.38 | 1.46 | 0.19 | 0.57 | 0.00 | |
| **Residence (ref.: rural)** |  |  |  |  |  | |
| urban | 0.07 | 1.07 | -0.09 | 0.22 | 0.40 | |
| **Family size (ref.: 1 person)** |  |  |  |  |  | |
| 2 persons | 0.07 | 1.08 | -0.26 | 0.41 | 0.67 | |
| 3-4 persons | 0.05 | 1.05 | -0.28 | 0.39 | 0.76 | |
| 5+ persons | 0.00 | 1.00 | -0.37 | 0.36 | 0.99 | |
| **Need factors** |  |  |  |  |  | |
| **Self-rated health (ref.: excellent)** |  |  |  |  |  | |
| **very good** | -0.22 | 0.80 | -0.61 | 0.17 | 0.27 | |
| good | -0.05 | 0.95 | -0.34 | 0.23 | 0.71 | |
| fair | -0.27 | 0.77 | -0.59 | 0.06 | 0.11 | |
| poor | -0.53 | 0.59 | -0.86 | -0.21 | 0.00 | |
| **Perceived symptom severity (ref.: not serious)** |  |  |  |  |  | |
| fair | -0.59 | 0.56 | -0.71 | -0.47 | 0.00 | |
| serious | -1.22 | 0.29 | -1.38 | -1.06 | 0.00 | |
| **Number of chronic diseases (ref.: 0)** |  |  |  |  |  | |
| 1 | -0.75 | 0.47 | -0.89 | -0.61 | 0.00 | |
| 2+ | -1.12 | 0.33 | -1.35 | -0.89 | 0.00 | |
| **Survey year** |  |  |  |  |  | |
| **Year (ref.: 2012)** |  |  |  |  |  | |
| 2014 | -0.26 | 0.77 | -0.39 | -0.12 | 0.00 | |
| 2016 | -0.39 | 0.68 | -0.54 | -0.24 | 0.00 | |
| 2018 | -0.45 | 0.64 | -0.60 | -0.30 | 0.00 | |
| 2020 | 0.19 | 1.20 | 0.02 | 0.35 | 0.02 | |
| 2022 | 0.38 | 1.46 | 0.20 | 0.56 | 0.00 | |
| Constant |  |  |  |  |  | |
| (_cons) | 1.64 | 5.17 | 0.71 | 2.58 | 0.00 | |

**Supplementary Table S4: Age–Sex Standardized Prevalence of Unmet Healthcare Needs among Chinese Residents, 2012–2022**

| **Year** | **N (unweighted)** | **N (weighted)** | **Standardized %** | **95% CI**  **(standardized)** |
| --- | --- | --- | --- | --- |
| 2012 | 4462 | 4388 | 37.50 | (34.59 - 40.41) |
| 2014 | 6024 | 5813 | 32.43 | (29.91 - 34.94) |
| 2016 | 6295 | 6029 | 28.94 | (26.29 - 31.589) |
| 2018 | 6395 | 6078 | 30.58 | (27.939 - 33.23) |
| 2020 | 4103 | 3866 | 40.05 | (36.81 - 43.29) |
| 2022 | 3646 | 3413 | 46.07 | (42.14 - 49.99) |

**Supplementary Table S5: Wave-to-wave transitions of the analytic sample, CFPS 2012–2022**

| **Transition component** | **2012 to 2014** | **2014 to 2016** | **2016 to 2018** | **2018 to 2020** | **2020 to 2022** |
| --- | --- | --- | --- | --- | --- |
| Analytic sample at preceding wave, N | 4462 | 6024 | 6295 | 6395 | 4103 |
| Retained in current wave, n (%) | 2507 (56.2%) | 3350 (55.6%) | 3506 (55.7%) | 2348 (36.7%) | 1710 (41.7%) |
| Lost: survey non-participation, n (%) | 230 (5.2%) | 434 (7.2%) | 747 (11.9%) | 1936 (30.3%) | 1011 (24.6%) |
| Lost: interviewed but no two-week illness reported, n (%) | 1553 (34.8%) | 2160 (35.9%) | 1984 (31.5%) | 1988 (31.1%) | 1311 (32.0%) |
| Lost: illness reported but missing outcome/covariates, n (%) | 172 (3.9%) | 80 (1.3%) | 58 (0.9%) | 123 (1.9%) | 71 (1.7%) |
| Newly entered analytic sample, n (%) | 3517 (58.4%) | 2945 (46.8%) | 2889 (45.2%) | 1755 (42.8%) | 1936 (53.1%) |
| of which: new CFPS respondents | 524 | 453 | 495 | 309 | 624 |
| of which: continuing respondents newly reporting illness | 2993 | 2492 | 2394 | 1446 | 1312 |
| Analytic sample at current wave, N | 6024 | 6295 | 6395 | 4103 | 3646 |

Note:Percentages for “retained” and the three “lost” categories use the preceding-wave analytic sample as the denominator; percentages for “newly entered” use the current-wave analytic sample as the denominator. For each wave pair: N(preceding) = retained + three lost categories; N(current) = retained + newly entered. The 2020 CFPS wave was administered primarily by telephone due to the COVID-19 pandemic.

**Supplementary Table S6: Preceding-wave characteristics of stayers and attriters, CFPS 2018-2020**

| **Characteristic at preceding wave** | **Stayers (n=2348)** | **Attriters (n=4047)** | **P value** | **SMD** |
| --- | --- | --- | --- | --- |
| **Healthcare need:met** | 1883(80.20%) | 3222(79.61%) | 0.577 | 0.015 |
| **Predisposing factors** |  |  |  |  |
| **Gender: female** | 1425 (60.69%) | 2432 (60.09%) | 0.615 | 0.013 |
| **Age** |  |  | <0.001 | 0.128 |
| 16-25 | 58 (2.47%) | 84 (2.08%) |  |  |
| 26-35 | 285 (12.14%) | 436 (10.77%) |  |  |
| 36-45 | 330 (14.05%) | 496 (12.26%) |  |  |
| 46-55 | 683 (29.09%) | 968 (23.92%) |  |  |
| 56-65 | 562 (23.94%) | 969 (23.94%) |  |  |
| 66+ | 430 (18.31%) | 1094 (27.03%) |  |  |
| **Marital status** |  |  | <0.001 | 0.091 |
| never married | 108 (4.60%) | 152 (3.76%) |  |  |
| married (having a spouse) | 2010 (85.60%) | 3349 (82.75%) |  |  |
| cohabitation | 14 (0.60%) | 22 (0.54%) |  |  |
| divorced | 39 (1.66%) | 96 (2.37%) |  |  |
| widowed | 177 (7.54%) | 428 (10.58%) |  |  |
| **Education** |  |  | <0.001 | 0.094 |
| primary school or below | 1285 (54.73%) | 2437 (60.22%) |  |  |
| junior high school | 607 (25.85%) | 916 (22.63%) |  |  |
| senior high school/secondary school/technical school/vocational senior school | 256 (10.90%) | 409 (10.11%) |  |  |
| 3-year college | 109 (4.64%) | 163 (4.03%) |  |  |
| 4-year college or above | 91 (3.88%) | 122 (3.01%) |  |  |
| **Household registration status: Agricultural** | 1795 (76.45%) | 3086 (76.25%) | 0.860 | 0.005 |
| **Employ: Non-employed (unemployed/not in labor force)** | 626 (26.66%) | 1253 (30.96%) | <0.001 | 0.095 |
| **Health beliefs** |  |  | 0.370 | 0.026 |
| very low | 95 (4.05%) | 193 (4.77%) |  |  |
| low | 370 (15.76%) | 653 (16.14%) |  |  |
| fair | 1199 (51.06%) | 2084 (51.49%) |  |  |
| high | 684 (29.13%) | 1117 (27.60%) |  |  |
| **Trust in doctors** |  |  | 0.324 | 0.036 |
| low | 281 (11.97%) | 449 (11.09%) |  |  |
| fair | 845 (35.99%) | 1419 (35.06%) |  |  |
| high | 1222 (52.04%) | 2179 (53.84%) |  |  |
| **Healthcare service satisfaction** |  |  | 0.395 | 0.032 |
| very unsatisfied | 55 (2.34%) | 81 (2.00%) |  |  |
| unsatisfied | 262 (11.16%) | 434 (10.72%) |  |  |
| fair | 524 (22.32%) | 904 (22.34%) |  |  |
| satisfied | 1280 (54.51%) | 2282 (56.39%) |  |  |
| very satisfied | 227 (9.67%) | 346 (8.55%) |  |  |
| **Enabling factors** |  |  |  |  |
| **Per Capita Household Income Percentile** |  |  | 0.192 | 0.035 |
| low | 728 (31.01%) | 1271 (31.41%) |  |  |
| low-middle | 684 (29.13%) | 1149 (28.39%) |  |  |
| middle-high | 497 (21.17%) | 933 (23.05%) |  |  |
| high | 439 (18.70%) | 694 (17.15%) |  |  |
| **Health insurance type** |  |  | 0.085 | 0.036 |
| Uninsured | 139 (5.92%) | 302 (7.46%) |  |  |
| Public medical insurance | 55 (2.34%) | 87 (2.15%) |  |  |
| Urban employee Basic Medical Insurance | 311 (13.25%) | 495 (12.23%) |  |  |
| Basic medical insurance for urban and rural residents | 1843 (78.49%) | 3163 (78.16%) |  |  |
| **Region** |  |  | 0.009 | 0.063 |
| Eastern China | 619 (26.36%) | 1223 (30.22%) |  |  |
| Central China | 525 (22.36%) | 891 (22.02%) |  |  |
| Western China | 860 (36.63%) | 1388 (34.30%) |  |  |
| Northeastern China | 344 (14.65%) | 545 (13.47%) |  |  |
| **Residence: rural** | 1280 (54.51%) | 2210 (54.61%) | 0.942 | 0.002 |
| **Familysize** |  |  | 0.258 | 0.033 |
| 1 | 105 (4.47%) | 211 (5.21%) |  |  |
| 2 | 498 (21.21%) | 892 (22.04%) |  |  |
| 3-4 | 836 (35.60%) | 1358 (33.56%) |  |  |
| 5+ | 909 (38.71%) | 1586 (39.19%) |  |  |
| **Need factors** |  |  |  |  |
| **Self-rated health** |  |  | <0.001 | 0.085 |
| excellent | 73 (3.11%) | 149 (3.68%) |  |  |
| very good | 94 (4.00%) | 197 (4.87%) |  |  |
| good | 690 (29.39%) | 1302 (32.17%) |  |  |
| fair | 394 (16.78%) | 726 (17.94%) |  |  |
| poor | 1097 (46.72%) | 1673 (41.34%) |  |  |
| **Perceived symptom severity** |  |  | 0.071 | 0.041 |
| not serious | 310 (13.20%) | 619 (15.30%) |  |  |
| fair | 1000 (42.59%) | 1694 (41.86%) |  |  |
| serious | 1038 (44.21%) | 1734 (42.85%) |  |  |
| **Number of diagnosed chronic disease** |  |  | <0.001 | 0.100 |
| 0 | 1397 (59.50%) | 2603 (64.32%) |  |  |
| 1 | 438 (18.65%) | 694 (17.15%) |  |  |
| 2+ | 513 (21.85%) | 750 (18.53%) |  |  |

**Supplementary Table S7: Unweighted vs IPW-weighted estimates from multivariable GEE models**

| **Variable** | **Unweighted OR (95%CI)** | **P** | **IPW-weighted OR (95%CI)** | **P** | **OR change %** |
| --- | --- | --- | --- | --- | --- |
| Constant | 3.745 (2.523-5.560) | <0.001 | 3.691 (2.477-5.501) | <0.001 | -1.5 |
| Gender: male (ref: female) | 0.957 (0.887-1.032) | 0.251 | 0.948 (0.878-1.024) | 0.172 | -0.9 |
| Age 26-35 (ref: 16-25) | 0.859 (0.719-1.027) | 0.095 | 0.872 (0.729-1.045) | 0.137 | 1.5 |
| Age 36-45 | 0.790 (0.656-0.951) | 0.013 | 0.790 (0.655-0.953) | 0.014 | 0.1 |
| Age 46-55 | 0.704 (0.585-0.848) | <0.001 | 0.709 (0.588-0.856) | <0.001 | 0.7 |
| Age 56-65 | 0.573 (0.474-0.693) | <0.001 | 0.579 (0.478-0.701) | <0.001 | 1 |
| Age 66+ | 0.522 (0.427-0.639) | <0.001 | 0.526 (0.429-0.645) | <0.001 | 0.7 |
| Married (ref: never married) | 0.790 (0.673-0.927) | 0.004 | 0.785 (0.669-0.923) | 0.003 | -0.5 |
| Cohabitation | 1.046 (0.678-1.615) | 0.838 | 1.033 (0.666-1.602) | 0.886 | -1.3 |
| Divorced | 0.814 (0.641-1.034) | 0.093 | 0.818 (0.641-1.044) | 0.107 | 0.4 |
| Widowed | 0.893 (0.731-1.091) | 0.269 | 0.894 (0.730-1.095) | 0.279 | 0.1 |
| Junior high (ref: primary or below) | 1.017 (0.939-1.101) | 0.682 | 1.017 (0.939-1.102) | 0.676 | 0.1 |
| Senior high/secondary/technical | 1.176 (1.056-1.310) | 0.003 | 1.176 (1.055-1.311) | 0.003 | 0 |
| 3-year college | 1.340 (1.135-1.582) | <0.001 | 1.330 (1.123-1.575) | <0.001 | -0.7 |
| 4-year college or above | 1.528 (1.274-1.832) | <0.001 | 1.516 (1.262-1.821) | <0.001 | -0.8 |
| Hukou: non-agricultural (ref: agricultural) | 1.110 (1.014-1.215) | 0.024 | 1.096 (1.000-1.201) | 0.049 | -1.2 |
| Employed (ref: non-employed) | 1.171 (1.082-1.267) | <0.001 | 1.177 (1.086-1.274) | <0.001 | 0.5 |
| Health beliefs: low (ref: very low) | 0.856 (0.749-0.978) | 0.022 | 0.860 (0.751-0.985) | 0.029 | 0.5 |
| fair | 0.701 (0.613-0.802) | <0.001 | 0.701 (0.611-0.803) | <0.001 | 0 |
| high | 0.577 (0.499-0.668) | <0.001 | 0.571 (0.492-0.662) | <0.001 | -1.1 |
| Trust in doctors: fair (ref: low) | 0.796 (0.726-0.873) | <0.001 | 0.806 (0.734-0.884) | <0.001 | 1.2 |
| high | 0.745 (0.679-0.816) | <0.001 | 0.750 (0.683-0.823) | <0.001 | 0.7 |
| Satisfaction: unsatisfied (ref: very unsatisfied) | 1.010 (0.804-1.269) | 0.929 | 0.997 (0.793-1.252) | 0.976 | -1.4 |
| fair | 0.969 (0.778-1.207) | 0.782 | 0.950 (0.762-1.183) | 0.645 | -2 |
| satisfied | 0.823 (0.662-1.023) | 0.079 | 0.812 (0.653-1.009) | 0.061 | -1.3 |
| very satisfied | 0.735 (0.578-0.934) | 0.012 | 0.722 (0.568-0.918) | 0.008 | -1.7 |
| Income: low-middle (ref: low) | 0.967 (0.897-1.042) | 0.381 | 0.971 (0.900-1.047) | 0.441 | 0.4 |
| middle-high | 0.982 (0.905-1.065) | 0.657 | 0.978 (0.902-1.062) | 0.602 | -0.3 |
| high | 1.022 (0.930-1.123) | 0.655 | 1.022 (0.929-1.125) | 0.652 | 0 |
| Public medical insurance (ref: uninsured) | 0.838 (0.673-1.043) | 0.114 | 0.853 (0.683-1.065) | 0.161 | 1.8 |
| UEBMI | 0.896 (0.784-1.025) | 0.111 | 0.905 (0.790-1.037) | 0.150 | 1 |
| URRBMI | 0.831 (0.747-0.924) | <0.001 | 0.826 (0.743-0.920) | <0.001 | -0.5 |
| Central China (ref: Eastern) | 0.884 (0.812-0.963) | 0.005 | 0.877 (0.805-0.957) | 0.003 | -0.8 |
| Western China | 0.916 (0.844-0.993) | 0.034 | 0.906 (0.834-0.984) | 0.019 | -1.1 |
| Northeastern China | 1.493 (1.357-1.643) | <0.001 | 1.486 (1.349-1.636) | <0.001 | -0.5 |
| Urban (ref: rural) | 1.220 (1.138-1.307) | <0.001 | 1.220 (1.138-1.308) | <0.001 | 0 |
| Family size 2 (ref: 1) | 1.037 (0.881-1.221) | 0.660 | 1.060 (0.898-1.251) | 0.494 | 2.2 |
| 3-4 | 0.938 (0.800-1.099) | 0.426 | 0.959 (0.816-1.127) | 0.613 | 2.3 |
| 5+ | 0.893 (0.760-1.049) | 0.169 | 0.906 (0.769-1.068) | 0.241 | 1.5 |
| SRH: very good (ref: excellent) | 1.066 (0.890-1.277) | 0.487 | 1.085 (0.904-1.303) | 0.379 | 1.8 |
| good | 1.063 (0.910-1.242) | 0.440 | 1.083 (0.925-1.267) | 0.322 | 1.9 |
| fair | 0.987 (0.840-1.159) | 0.871 | 0.998 (0.848-1.175) | 0.984 | 1.2 |
| poor | 0.788 (0.672-0.924) | 0.003 | 0.798 (0.680-0.938) | 0.006 | 1.3 |
| Severity: fair (ref: not serious) | 0.630 (0.587-0.675) | <0.001 | 0.630 (0.587-0.676) | <0.001 | 0 |
| serious | 0.339 (0.312-0.368) | <0.001 | 0.337 (0.310-0.367) | <0.001 | -0.6 |
| Chronic disease: 1 (ref: 0) | 0.452 (0.417-0.489) | <0.001 | 0.456 (0.421-0.495) | <0.001 | 1.1 |
| 2+ | 0.401 (0.364-0.441) | <0.001 | 0.406 (0.368-0.447) | <0.001 | 1.3 |
| Year 2014 (ref: 2012) | 0.752 (0.688-0.823) | <0.001 | 0.754 (0.689-0.824) | <0.001 | 0.2 |
| Year 2016 | 0.638 (0.582-0.700) | <0.001 | 0.647 (0.590-0.710) | <0.001 | 1.4 |
| Year 2018 | 0.603 (0.549-0.663) | <0.001 | 0.609 (0.555-0.669) | <0.001 | 1 |
| Year 2020 | 1.152 (1.043-1.272) | 0.005 | 1.156 (1.046-1.278) | 0.005 | 0.3 |
| Year 2022 | 1.379 (1.241-1.531) | <0.001 | 1.361 (1.224-1.513) | <0.001 | -1.3 |

**Sources of questionnaire items and variable standardization**

The original questionnaire items for some variables have been aligned with the variable definitions as shown below.

(1) **Marital status**

Based on the responses to the survey question “What is your current marital status?”, the categories are as follows: 1=Never married, 2=Married (having a spouse), 3=Cohabitation, 4=Divorced, 5=Widowed.

(2) **Household registration status (hukou)**

Based on the responses to the survey question “Your current household registration type is:”, the categories are as follows: 0=Agricultural, 1=Non-agricultural (including resident hukou)

**Supplementary Table S8: Classification of Household Registration Types**

| Final Category | Corresponding Options (2012–2018) | Corresponding Options (2020–2022) |
| --- | --- | --- |
| 0 = Agricultural | 1. Agricultural | 1. Agricultural |
| 1 = Non-Agricultural(Including Residential) | 3. Non-agricultural | 4. Non-agricultural |
|  |  | 7. Residential |

Note: The "Resident Household Registration" option was introduced starting with the 2020 survey; this option was not available in previous surveys. Excludes 5. No household registration and 79. Not applicable (non-Chinese nationality).

(3) **Employment status**

**Supplementary Table S9: Classification of Employment status**

| Final Category | Corresponding Options (2012) | Corresponding Options (2014–2022) |
| --- | --- | --- |
| 0=Non-employed (unemployed/not in labor force) | 0.unemployed | 0.unemployed  3.not in labor force |
| 1=Employed | 1.Employed | 1.Employed |

Note: Excludes 2012 8.Unable to determine whether the individual is unemployed or part of the economically inactive population (short questionnaire).

(4) **Health beliefs**

This section consists of three components: smoking, alcohol consumption, and physical exercise. For the smoking component, based on the response to the questionnaire question “Did you smoke cigarettes in the past month?”, scores were assigned as follows: 0 points = No, 1 point = Yes. For the alcohol consumption component, based on the questionnaire question “Did you drink alcohol at least 3 times a week in the past month?”, scores were assigned as follows: 0 points = No, 1 point = Yes. The physical activity section is based on the 2012 questionnaire question, “In the most recent month when you were not on vacation, how often did you participate in physical exercise?”; the 2014, 2016, and 2018 questionnaire questions, “Including physical education, how often did you participate in physical exercise in the past week?”; and the 2020 and 2022 questionnaire question: “in the past 12 months, how often did you participate in physical fitness and leisure activities?” Since the response options differed slightly between 2012, 2014–2018, and 2022, the classification method is shown in the table below:

**Supplementary Table S10: Classification of Physical Exercise**

| **Final Category** | **Corresponding Options (2012)** | **Corresponding Options (2014-2018)** | **Corresponding Options (2020-2022)** |
| --- | --- | --- | --- |
| 0=Irregular exercise | 3. 2-3 times a month  4. Once a month  5. Never | 3 or more times a week | 1. Less than once a month  2. More than once a month, but less than once a week  3. 1 to 2 times a week  8. Never |
| 1=Regular exercise | 1.Almost daily  2.2-3 times a week | Less than three times a week | 4. 3 to 4 times a week  5. More than 5 times a week  6. Once a day  7. Twice a day or more |

The sum of the scores from the three sections constitutes the health belief score, where 1 = 0 points (very low), 2 = 1 point (low), 3 = 2 points (moderate), and 4 = 3 points (high).

(5) **Trust in doctors**

Based on the responses to the survey question “How much do you trust doctors?”, which used an 11-point scale ranging from 0 to 10—where 0 indicates “very little trust” and 10 indicates “a great deal of trust”—the scores were categorized as follows for subsequent analysis: 0–3, 4–6, and 7–10 were classified as 1 = low, 2 = moderate, and 3 = high.

(6) **Medical service satisfaction**

Based on the responses to the survey question “Are you satisfied with the overall medical service at the healthcare facility?”, the responses were categorized as follows: 1 = Very dissatisfied, 2 = Dissatisfied, 3 = Neutral, 4 = Satisfied, and 5 = Very satisfied.

(7) **Health insurance type**

Based on the responses to the survey question “Do you have any of the following medical insurances?”, the classification is shown in the table below:

**Supplementary Table S11: Classification of Health Insurance**

| **Final Category** | **Corresponding Options (2012-2018)** | **Corresponding Options (2020-2022)** |
| --- | --- | --- |
| 1=Uninsured | 78. None of the above | 78. None of the above |
| 2=Public medical insurance | 1. Public medical insurance | 1. Public medical insurance |
| 3=Urban employee Basic Medical Insurance | 2. Urban Employee Basic Medical Insurance | 2. Urban Employee Basic Medical Insurance |
| 4=Basic medical insurance for urban and rural residents (including Urban Resident Basic Medical Insurance and New Rural Cooperative Medical Insurance) | 3. Urban Resident Basic Medical Insurance | 3. Urban Resident Basic Medical Insurance |
|  | 4. New Rural Cooperative Medical Insurance | 5. New Rural Cooperative Medical Insurance |
|  | - | 6. Basic medical insurance for urban and rural residents |

Note: Excluding samples that selected only Option 4 (Supplementary Medical Insurance), the priority order for each insurance type is: Public medical insurance > Urban Employee Basic Medical Insurance > Basic medical insurance for urban and rural residents.

(8) **Region**

Based on China’s four major economic regions, the classification is as shown in the table below:

**Supplementary Table S12: Classification of Region**

| **Final Category** | **Included Provincial-level Administrative Divisions** |
| --- | --- |
| 1=Eastern China | Beijing Municipality, Tianjin Municipality, Hebei Province, Shanghai Municipality, Jiangsu Province, Zhejiang Province, Fujian Province, Shandong Province, Guangdong Province, Hainan Province |
| 2=Central China | Shanxi Province, Anhui Province, Jiangxi Province, Henan Province, Hubei Province, Hunan Province |
| 3=Western China | Inner Mongolia Autonomous Region, Guangxi Zhuang Autonomous Region, Chongqing Municipality, Sichuan Province, Guizhou Province, Yunnan Province, Tibet Autonomous Region, Shaanxi Province, Gansu Province, Qinghai Province, Ningxia Hui Autonomous Region, Xinjiang Uygur Autonomous Region |
| 4=Northeastern China | Liaoning Province, Jilin Province, Heilongjiang Province |

(9) **Self-rated health**

Based on the responses to the questionnaire question “How would you rate your health status?”, the answers were categorized as follows: 1 = Very healthy, 2 = Quite healthy, 3 = Fairly healthy, 4 = Average, and 5 = Unhealthy.

(10) **Perceived symptom severity**

Based on the responses to the questionnaire question “ How serious do you think the symptom is?”, the answers were categorized as follows: 1 = Not severe, 2 = Moderate, 3 = Severe.

(11) **Number of diagnosed chronic disease**

Based on the responses to the questionnaire items “During the past six months, have you had any doctor-diagnosed chronic disease?” and “What was your doctor’s diagnosis of the disease you suffered from?”, the data was categorized as follows: 1 = 0 diseases, 2 = 1 disease, 3 = 2 or more diseases. Samples that indicated having a chronic disease but did not specify the specific condition were treated as missing values and were excluded.

**Supplementary Table S13: Variable Coding**

| **Variable Type** | **Variable Name** | **Coding Scheme** | **Variable label** |
| --- | --- | --- | --- |
| **Dependent variable** | Healthcare needs | 0 = met，1 = unmet | qp304 |
| **Independent variables** |  |  |  |
| **Predisposing factors** | Gender | 0 = female，1 = male | cfps_gender |
|  | Age | 1 = 16-25, 2 = 26-35, 3 = 36-45, 4 = 46-55, 5 = 56-65, 6 = 66 or obove | cfps_age |
|  | Marital status | 1 = never married, 2 = married (having a spouse), 3 = cohabitation, 4 = divorced, 5 = widowed | qe104(2012)  qea0(2014 - 2022) |
|  | Education | 1 = primary school or below, 2 = junior high school, 3 = senior high school/secondary school/technical school/vocational senior school, 4 = 3-year college, 5 = 4-year college or above | cfps*edu(cfps12edu for 2012) |
|  | Household registration status (hukou) | 0=Agricultural, 1=Non-agricultural (including resident hukou) | qa301 |
|  | Employment status | 0=Non-employed (unemployed/not in labor force), 1=Employed | EMPLOY |
|  | Health beliefs | 1 = very low, 2 = low, 3 = fair, 4 = high | derived from qq201, qq301, qp701 |
|  | Trust in doctors | 1 = low, 2 = fair, 3 = high | qn10026 |
|  | Medical service satisfaction | 1 = very unsatisfied, 2 = unsatisfied, 3 = fair, 4 = satisfied, 5 = very satisfied | qp602 |
| **Enabling factors** | Per capita household income percentile | 1 = low, 2 = low-middle, 3 = middle-high, 4 = high | fincperadj_p(2012)  fincome1_per_p(2014 - 2022) |
|  | Health insurance type | 1=Uninsured, 2=Public medical insurance, 3=Urban employee Basic Medical Insurance, 4=Basic medical insurance for urban and rural residents | qp605 |
|  | Region | 1 = Eastern China, 2 = Central China, 3 = Western China, 4 = Northeastern China | provcd*(provcd12 for 2012) |
|  | Residence | 0 = rural, 1 = urban | urban*(urban12 for 2012) |
|  | Familysize(members) | 1=1, 2=2, 3=3 – 4, 4=5 or more | familysize |
| **Need factors** | Self-rated health | 1 = excellent, 2 = very good, 3 = good, 4 = fair, 5 = poor | qp201 |
|  | Perceived symptom severity | 1 = not serious, 2 = fair, 3 = serious | qp303 |
|  | Number of diagnosed chronic disease | 1 = 0, 2 = 1, 3= 2 or more | qp401, qp402A, qp402B |

**Supplementary Table S14: Intra-group Variation**

| **variable** | **pid_changed** | **pid_unchanged** | **pid_pct%** |
| --- | --- | --- | --- |
| Healthcare needs | 4569 | 6498 | 41.28490106 |
| Gender | 0 | 11067 | 0 |
| Age | 5291 | 5776 | 47.80880094 |
| Marital status | 903 | 10164 | 8.159392789 |
| Education | 665 | 10402 | 6.008855155 |
| Household registration status (hukou) | 598 | 10469 | 5.403451703 |
| Employment status | 2791 | 8276 | 25.21911991 |
| Health beliefs | 5705 | 5362 | 51.54965212 |
| Trust in doctors | 6228 | 4839 | 56.27541339 |
| Medical service satisfaction | 7309 | 3758 | 66.04319147 |
| Per capita household income percentile | 7247 | 3820 | 65.48296738 |
| Health insurance type | 2710 | 8357 | 24.48721424 |
| Region | 244 | 10823 | 2.204752869 |
| Residence | 987 | 10080 | 8.918406072 |
| Familysize(members) | 3978 | 7089 | 35.94470046 |
| Self-rated health | 6760 | 4307 | 61.08249752 |
| Perceived symptom severity | 7109 | 3958 | 64.23601699 |
| Number of diagnosed chronic disease | 5743 | 5324 | 51.89301527 |

Note: Clogit automatically excludes individuals with no variation in the outcome; there are 13,697 observations and 4,569 individuals.

**Supplementary Table S15: Collinearity Diagnosis**

|  | **GVIF** | **Df** | **GVIF^(1/(2*Df))** |
| --- | --- | --- | --- |
| factor(Age) | 2.538635 | 5 | 1.09764 |
| factor(Marital status) | 1.931295 | 4 | 1.085753 |
| factor(Education) | 2.063538 | 4 | 1.094779 |
| factor(Household registration status) | 1.927184 | 1 | 1.388231 |
| factor(Employment status) | 1.373201 | 1 | 1.171837 |
| factor(Health beliefs) | 1.110699 | 3 | 1.017652 |
| factor(Trust in doctors) | 1.091679 | 2 | 1.022171 |
| factor(Medical service satisfaction) | 1.210847 | 4 | 1.024203 |
| factor(Per capita household income percentile) | 1.496594 | 3 | 1.069508 |
| factor(Health insurance type) | 1.812288 | 3 | 1.104175 |
| factor(Region) | 1.215407 | 3 | 1.033047 |
| factor(Residence) | 1.390451 | 1 | 1.179174 |
| factor(Familysize) | 1.584636 | 3 | 1.079746 |
| factor(Self-rated health) | 1.388527 | 4 | 1.041884 |
| factor(Perceived symptom severity) | 1.246094 | 2 | 1.056544 |
| factor(Number of diagnosed chronic disease) | 1.182244 | 2 | 1.042742 |
| factor(Year) | 1.264461 | 5 | 1.023742 |

**Supplementary Table S16: Urban-Rural Year Interaction**

| **Interaction (ref: rural, vs 2012)** | **OR** | **95%CI low** | **95%CI high** | **P value** |
| --- | --- | --- | --- | --- |
| year 2014 × urban | 1.307 | 1.095 | 1.56 | 0.003 |
| year 2016 × urban | 1.203 | 1.003 | 1.444 | 0.047 |
| year 2018 × urban | 1.229 | 1.023 | 1.476 | 0.028 |
| year 2020 × urban | 1.383 | 1.14 | 1.677 | 0.001 |
| year 2022 × urban | 1.346 | 1.101 | 1.645 | 0.004 |

**Supplementary Table S17: Sensitivity analysis of GEE with AR(1) correlation structure**

| **Term** | **OR[AR(1)]** | **CI_low** | **CI_high** | **P** |
| --- | --- | --- | --- | --- |
| Intercept | 3.7015 | 2.4858 | 5.5119 | <0.001 |
| C(Gender)[T.1] | 0.9584 | 0.888 | 1.0344 | 0.276 |
| C(Age)[T.2] | 0.8625 | 0.7217 | 1.0307 | 0.104 |
| C(Age)[T.3] | 0.7914 | 0.6568 | 0.9537 | 0.014 |
| C(Age)[T.4] | 0.701 | 0.5817 | 0.8448 | <0.001 |
| C(Age)[T.5] | 0.5694 | 0.4707 | 0.6886 | <0.001 |
| C(Age)[T.6] | 0.5229 | 0.4269 | 0.6405 | <0.001 |
| C(Marital status)[T.2] | 0.8007 | 0.6807 | 0.9419 | 0.007 |
| C(Marital status)[T.3] | 1.0817 | 0.6973 | 1.6781 | 0.726 |
| C(Marital status)[T.4] | 0.8268 | 0.6505 | 1.051 | 0.12 |
| C(Marital status)[T.5] | 0.9142 | 0.7468 | 1.1191 | 0.384 |
| C(Education)[T.2] | 1.0278 | 0.9492 | 1.1129 | 0.499 |
| C(Education)[T.3] | 1.183 | 1.0616 | 1.3182 | 0.002 |
| C(Education)[T.4] | 1.3441 | 1.1367 | 1.5893 | <0.001 |
| C(Education)[T.5] | 1.5352 | 1.2802 | 1.8409 | <0.001 |
| C(Household registration status)[T.1] | 1.1106 | 1.0139 | 1.2166 | 0.024 |
| C(Employment status)[T.1] | 1.1735 | 1.0841 | 1.2702 | <0.001 |
| C(Health beliefs)[T.2] | 0.8595 | 0.7515 | 0.983 | 0.027 |
| C(Health beliefs)[T.3] | 0.7015 | 0.6127 | 0.8031 | <0.001 |
| C(Health beliefs)[T.4] | 0.5742 | 0.4956 | 0.6653 | <0.001 |
| C(Trust in doctors)[T.2] | 0.8054 | 0.7343 | 0.8833 | <0.001 |
| C(Trust in doctors)[T.3] | 0.753 | 0.6864 | 0.8259 | <0.001 |
| C(Medical service satisfaction)[T.2] | 1.0213 | 0.8103 | 1.2872 | 0.859 |
| C(Medical service satisfaction)[T.3] | 0.9746 | 0.7801 | 1.2176 | 0.821 |
| C(Medical service satisfaction)[T.4] | 0.8244 | 0.6611 | 1.0282 | 0.087 |
| C(Medical service satisfaction)[T.5] | 0.7303 | 0.5724 | 0.9316 | 0.011 |
| C(Per capita household income percentile))[T.2] | 0.9664 | 0.8961 | 1.0421 | 0.374 |
| C(Per capita household income percentile))[T.3] | 0.9775 | 0.9012 | 1.0603 | 0.583 |
| C(Per capita household income percentile))[T.4] | 1.0206 | 0.9283 | 1.1221 | 0.674 |
| C(Health insurance type)[T.2] | 0.8292 | 0.6656 | 1.033 | 0.095 |
| C(Health insurance type)[T.3] | 0.8852 | 0.7731 | 1.0135 | 0.077 |
| C(Health insurance type)[T.4] | 0.8251 | 0.7417 | 0.9179 | <0.001 |
| C(Region)[T.2] | 0.8831 | 0.8105 | 0.9622 | 0.005 |
| C(Region)[T.3] | 0.9141 | 0.8425 | 0.9919 | 0.031 |
| C(Region)[T.4] | 1.5021 | 1.3639 | 1.6543 | <0.001 |
| C(Residence)[T.1] | 1.2186 | 1.1369 | 1.3062 | <0.001 |
| C(Familysize)[T.2] | 1.0363 | 0.88 | 1.2204 | 0.669 |
| C(Familysize)[T.3] | 0.9322 | 0.7949 | 1.0932 | 0.388 |
| C(Familysize)[T.4] | 0.8847 | 0.7522 | 1.0405 | 0.139 |
| C(Self-rated health)[T.2] | 1.0651 | 0.8892 | 1.2759 | 0.493 |
| C(Self-rated health)[T.3] | 1.0638 | 0.9102 | 1.2433 | 0.437 |
| C(Self-rated health)[T.4] | 0.9837 | 0.837 | 1.156 | 0.841 |
| C(Self-rated health)[T.5] | 0.7905 | 0.6738 | 0.9275 | 0.004 |
| C(Perceived symptom severity)[T.2] | 0.6273 | 0.585 | 0.6728 | <0.001 |
| C(Perceived symptom severity)[T.3] | 0.3386 | 0.3116 | 0.368 | <0.001 |
| C(Number of diagnosed chronic disease)[T.2] | 0.4467 | 0.4119 | 0.4844 | <0.001 |
| C(Number of diagnosed chronic disease)[T.3] | 0.395 | 0.3586 | 0.4352 | <0.001 |
| C(Year)[T.2014] | 0.757 | 0.6922 | 0.8279 | <0.001 |
| C(Year)[T.2016] | 0.639 | 0.5825 | 0.7008 | <0.001 |
| C(Year)[T.2018] | 0.6001 | 0.546 | 0.6596 | <0.001 |
| C(Year)[T.2020] | 1.1421 | 1.0337 | 1.2619 | 0.009 |
| C(Year)[T.2022] | 1.3632 | 1.2265 | 1.515 | <0.001 |
